# Ferritinophagy activation states determine the susceptibility to ferroptosis of macrophages in bone marrow and spleen

**DOI:** 10.7150/ijbs.114545

**Published:** 2025-07-11

**Authors:** Xin Lai, Aimin Wu, Yao Liu, Chen Liu, Junzhou Chen, Ke Gu, Bing Yu, Hui Yan, Junqiu Luo, Ping Zheng, Jie Yu, Daiwen Chen

**Affiliations:** 1 Institute of Animal Nutrition, Sichuan Agricultural University, Chengdu, China.; 2 Key Laboratory for Animal Disease-resistance Nutrition of China Ministry of Education, Sichuan Agricultural University, Chengdu, China.

**Keywords:** spleen and bone marrow macrophages, ferroptosis, autophagic flux, ferritinophagy, NCOA4

## Abstract

Macrophages exhibit heterogeneity due to their presence in different tissues that have distinct cell fates. Ferroptosis is one type of cellular fate, but the sensitivity of different types of macrophages to ferroptosis and the associated molecular mechanisms are not clear. This study explored the ferroptosis sensitivity of bone marrow and splenic macrophage, focusing on the contribution of ferritinophagy. We found that bone marrow M2 macrophages were more susceptible to ferroptosis, which was attributed to their lower solute carrier family 40 member 1 (SLC40A1) and ferritin heavy/light chain (FTH/L) expression and higher labile iron levels compared to those of splenic macrophages. Further, ferritinophagy activation, particularly in M2 macrophages, was identified as the primary cause of increased labile iron levels, as evidenced by experiments using autophagic flux modifiers and RAW264.7 cells with autophagy related 5 (ATG5) and nuclear receptor coactivator 4 (NCOA4) knockdown and NCOA4 knockout. These results provide a new direction for further understanding the heterogeneity and functionality of macrophages, and offers innovative treatments for a variety of health issues in which macrophage regulation plays a critical role.

## Introduction

Macrophages are important cells in the immune system with various polarization states that influence their functions and roles in diseases 1, 2. The primary macrophage polarization types are M1 and M2. M1 macrophages, induced by IFN-γ and lipopolysaccharide (LPS), are pro-inflammatory, which contribute to host defenses by producing high levels of pro-inflammatory cytokines and reactive nitrogen and oxygen intermediates. M1 macrophages are typically associated with anti-tumor and anti-microbial responses 3, 4. M2 macrophages, induced by IL-4 and IL-13, participate in wound healing, tissue repair, and immune regulation. M2 macrophages produce anti-inflammatory cytokines and are associated with tumor progression and inflammatory response suppression 3, 4. The balance between M1 and M2 macrophages is critical for immune homeostasis, and dysregulation can lead to various pathologies, including chronic inflammation, cancer, and autoimmune diseases. To develop targeted therapies for these conditions, understanding the mechanisms governing macrophage polarization and function is crucial 5.

Iron profoundly shapes macrophage behavior, which influences macrophage polarization, inflammation, and disease outcomes. Through diverse mechanisms, iron levels dictate the macrophage response: high iron promotes anti-inflammatory M2 markers, while low iron fuels pro-inflammatory M1 responses 6. However, in a separate study, iron overload induced M1 polarization via reactive oxygen species (ROS) and p53 acetylation 7 This dichotomy in the influence of iron on macrophage activation underscores the complexity of its metabolic interactions and requires further investigation. Notably, the current literature has only minimally addressed the role of iron-induced ferroptosis in macrophage regulation, which is therefore an underexplored research area. Ferroptosis is a form of regulated cell death that is iron-dependent and linked to lipid peroxide accumulation 8. Given the critical involvement of spleen and bone marrow macrophages in iron handling and storage 9, these cells are potentially key sites where ferroptosis may impart significant physiological or pathological consequences; however, few studies have explored the differences in ferroptosis sensitivity among macrophages from different tissue sites 10.

In this study, we revealed distinct difference in resistance to high iron-induced ferroptosis among macrophage subtypes, with bone marrow M2 the most susceptible, followed by bone marrow M1, splenic M1, and splenic M2. Mechanistically, bone marrow M2 macrophages showed the highest ferroptosis susceptibility, which was linked to low solute carrier family 40 member 1** (**SLC40A1) and ferritin heavy/light chain (FTH/L) expression and led to increased labile iron. Ferritinophagy is the autophagic degradation of ferritin, a process that releases iron into cells. This process balances iron availability and can trigger ferroptosis 11. Activation of ferritinophagy, particularly in M2 macrophages, was identified as a key factor elevating labile iron levels, and was confirmed through autophagic flux modifiers, autophagy related 5 (ATG5) and nuclear receptor coactivator 4 (NCOA4) knockdown, and NCOA4 knockout in RAW264.7 cell experiments.

Our findings highlight critical differences in ferroptosis susceptibility between tissue-resident macrophages (macrophage heterogeneity), suggesting potential therapeutic avenues. Targeting ferroptosis mechanisms in specific macrophage populations may provide new strategies for treating iron-related disorders, inflammatory diseases, and cancers 12. Inducing ferroptosis in tumor-associated macrophages may enhance cancer therapy effectiveness 13, while inhibiting it in inflammatory macrophages may protect against tissue damage in chronic conditions 14.

In conclusion, this study provides a detailed analysis of the differential ferroptosis sensitivity between bone marrow and splenic macrophages, driven by iron metabolism, autophagy, and ferritinophagy. Our results identify new possibilities for manipulating macrophage functions through ferroptosis regulation, with significant implications for disease treatment.

## Material and Methods

### Animal model

The experimental procedures were authorized by the Institutional Animal Care and Use Committee of the Laboratory Animal Center at Sichuan Agricultural University (Approval No. SICAU-2015-033). Experiment 1: Twenty 6-week-old male C57BL/6 J mice (provided by Chengdu Dossy Experimental Animals Co., Ltd.) were randomly assigned to two groups with comparable body weights: control (50 mg/kg Fe, n=10) and high iron diet (5,000 mg/kg Fe, n=10). The experiment lasted for 5 weeks before the mice were sacrificed. Bone marrow from femurs and tibias were harvested, and bone marrow cells were isolated by centrifugation at 3,000 rpm. The isolated cells were then resuspended in Dulbecco's modified Eagle medium (DMEM) supplemented with 2% fetal bovine serum to prepare a single-cell suspension for subsequent flow cytometric analysis. Experiment 2: Twelve 6-week-old male C57BL/6 J mice (provided by Chengdu Dossy Experimental Animals Co., Ltd.) were randomly assigned to two groups with comparable body weights (approx. 24 g): control (n=6) and erastin injection group (n=6). Mice were intraperitoneal injected with 25 mg/kg body weight of erastin or solvent (10% Dimethyl sulfoxide + 40% Polyethylene glycol 300+ 5% Tween‑80 + 45% physiological saline) for 2 days at 12 h intervals. Mice were anesthetized 6 h after the last injection. Liver and lung tissues were enzymatically digested and filtered through a 70 µm strainer to obtain single cell suspensions enriched in tissue-resident macrophages.

### Chemicals and reagents

A full list of chemicals and reagents used in this study, including catalog numbers and suppliers, is provided in Supplementary Table S1.

### Bone marrow derived macrophages (BMDMs) isolation and stimulation

BMDMs were prepared as described previously 15. Briefly, bone marrow cells were extracted from the femurs and tibias of 6-8-week-old C57BL/6J mice and isolated via centrifugation. The cells were then cultured in DMEM supplemented with 10% fetal bovine serum (FBS), 1% penicillin-streptomycin (PS), and 20 ng/mL macrophage colony-stimulating factor (M-CSF) (GMP-TL654, T&L Biotechnology, Beijing, China) to promote macrophage differentiation over 7 days. After differentiation, adherent cells were collected and replated for downstream applications. For M1 polarization, BMDMs (2.5 × 10⁵ cells per well) were seeded in 12-well plates and stimulated with 100 ng/mL lipopolysaccharide (LPS) (L2880, Sigma-Aldrich, St. Louis, MO, USA) for 6 h. M2 polarization was achieved by treating cells with 20 ng/mL interleukin-4 (IL-4) (214-14, PeproTech, Rocky Hill, NJ, USA) for 24 h.

### Splenic macrophages isolation and stimulation

Spleen macrophages were prepared as previously described 16, 17. Macrophages were obtained from three donor spleens. To prepare splenic macrophages, splenocytes were homogenized and passed through a metal sieve to eliminate debris. After washing twice with warm RPMI medium containing 10% FBS, cells were cultured in M-CSF (20 ng/mL). Non-adherent cells were removed after 3 days by replacing the medium to enhance adherence. This process was repeated on day 6, and adherent cells were collected on day 7, yielding mature macrophages. Cells were harvested using 1× trypsin-EDTA (0.25%) (25-200-056, Gibco, Thermo Fisher Scientific, Waltham, MA, United States) at specified culture time points.

### RAW264.7 cell line culture

RAW264.7 monocytes were cultured in DMEM supplemented with 10% FBS and 1% PS at 37°C and 5% CO_2_. The cells were stimulated with mouse IFN-γ (2.5 ng/mL) and LPS (200 ng/mL) for 24 h to induce the M1 phenotype, or with mouse IL-4 (10 ng/mL) for 48 h to induce the M2 phenotype. Untreated RAW264.7 cells were used as the M0 phenotype.

### RAW264.7 NCOA4 knockout cell line

RAW264.7 NCOA4 knockout cells were generated using CRISPR/Cas9-based gene editing (see Supplementary Methods for detailed procedures).

### Real-time quantitative PCR

RNA samples were extracted from tissues and cells using TRIzol reagent (15596026, Invitrogen, Thermo Fisher Scientific, Waltham, MA, United States) and processed according to the manufacturer's instructions. The RNA concentration was normalized to 1 μg/μL. A reverse transcription kit (RR037B, Takara Bio, Kusatsu, Shiga, Japan) was used following the manufacturer's guidelines. The relative gene expression was calculated using the ΔΔCT method and normalized to the housekeeping gene *Actb*. The primer design is provided in Table S2.

### Western immunoblotting

Western blotting was performed following established protocols 18. The primary antibodies were diluted at a ratio of 1:1,000. The secondary antibodies, goat anti-rabbit and goat anti-mouse antibodies conjugated with HRP (sc-2030 and sc-2031, Santa Cruz Biotechnology, Dallas, TX, United States), were diluted to a concentration of 1:3,000. Detailed antibody information is provided in Table S3.

### Flow cytometry analysis of macrophage polarization

Fluorescently labeled antibodies (CD16/32, CD45, F4/80, CD11b, CD11c, CD86, and CD206) were used to label cells following the manufacturer's guidelines. Cellular samples were processed using a BD FACSVerse^TM^ instrument (BD Biosciences) and subsequently analyzed using FlowJo10 software. Detailed antibody specifications can be found in Table S2.

### Fluorescence staining with calcein-AM and propidium iodide

Cell viability and death were assessed using calcein-AM and PI staining (see Supplementary Methods for details).

### Measurements of lipid peroxidation and total ROS

To assess cellular ROS and lipid peroxidation levels, macrophages were subjected to treatment with 50 μM 2′,7′-Dichlorofluorescin diacetate (DCFH-DA) (D6883, Sigma-Aldrich, St. Louis, MO, United States) and 5 μM C11-BODIPY (D3861, Invitrogen, Thermo Fisher Scientific, Waltham, MA, United States), respectively, for 30 min at 37°C. Macrophages were then washed three times with PBS. The cell fluorescence intensity was analyzed using flow cytometry.

### Detection of intracellular labile ferrous ions (Fe^2+^)

FerroFarRed^TM^ (GC903-01, Goryo Chemical, Tokyo, Japan) was used in accordance with the guidelines provided by the manufacturer.

### Transcriptomic data acquisition and analysis

Total RNA was isolated using TRIzol reagent (15596026, Invitrogen, Thermo Fisher Scientific, Waltham, MA, United States). Detailed protocols for RNA library preparation and high-throughput sequencing are provided in Supplementary Methods.

### Autophagic flux monitoring using a dual-labeled adenovirus (mRFP-GFP-LC3)

Autophagic flux was monitored using an mRFP-GFP-LC3 dual-labeled adenoviral system, as described in Supplementary Methods.

### FTH1 knockdown by shRNA adenovirus transduction

FTH1 knockdown was achieved via shRNA adenoviral transduction. shRNA sequences and transduction protocols are detailed in Supplementary Methods.

### ATG5 and NCOA4 knockdown

Gene silencing of ATG5 and NCOA4 was performed using siRNA transfection with Lipofectamine 3000 (see Supplementary Methods for siRNA sequences and procedures).

### Transmission electron microscopy (TEM)

Cells were fixed in 2.5% glutaraldehyde, post-fixed in osmium tetroxide, dehydrated in ethanol, and embedded in resin. Ultrathin sections were stained with uranyl acetate and lead citrate and examined using a Hitachi HT7800 TEM at 80 kV. Digital images were captured for ultrastructural analysis.

### Single cell RNA sequencing data processing and macrophage subtype analysis

Single cell RNA sequencing datasets were obtained from the Gene Expression Omnibus (GEO) database, including normal spleen (GSE134355), splenic marginal zone lymphoma (SMZL) (GSE286927), acute myeloid leukemia (AML) (GSE120221), and its corresponding healthy control (GSE223844). Details of single-cell RNA sequencing data processing, integration, clustering, and macrophage subtype annotation are provided in Supplementary Methods.

### Statistical analysis

Flow cytometry data were analyzed using FlowJo (version 10.4). Microsoft Excel software and GraphPad Prism were used for statistical analyses, with all data presented as mean ± standard error of mean. Statistical comparisons between groups were performed using unpaired two-tailed Student's t test and one-way ANOVA (Tukey). Results with P ≤ 0.05 were deemed statistically significant (*P < 0.05, ** P < 0.01, *** P < 0.001). All graphical representations were generated using GraphPad Prism 7 software.

## Results

### Macrophage sensitivity to iron-induced ferroptosis is tissue-specific and polarization-dependent

**Figure 1:**
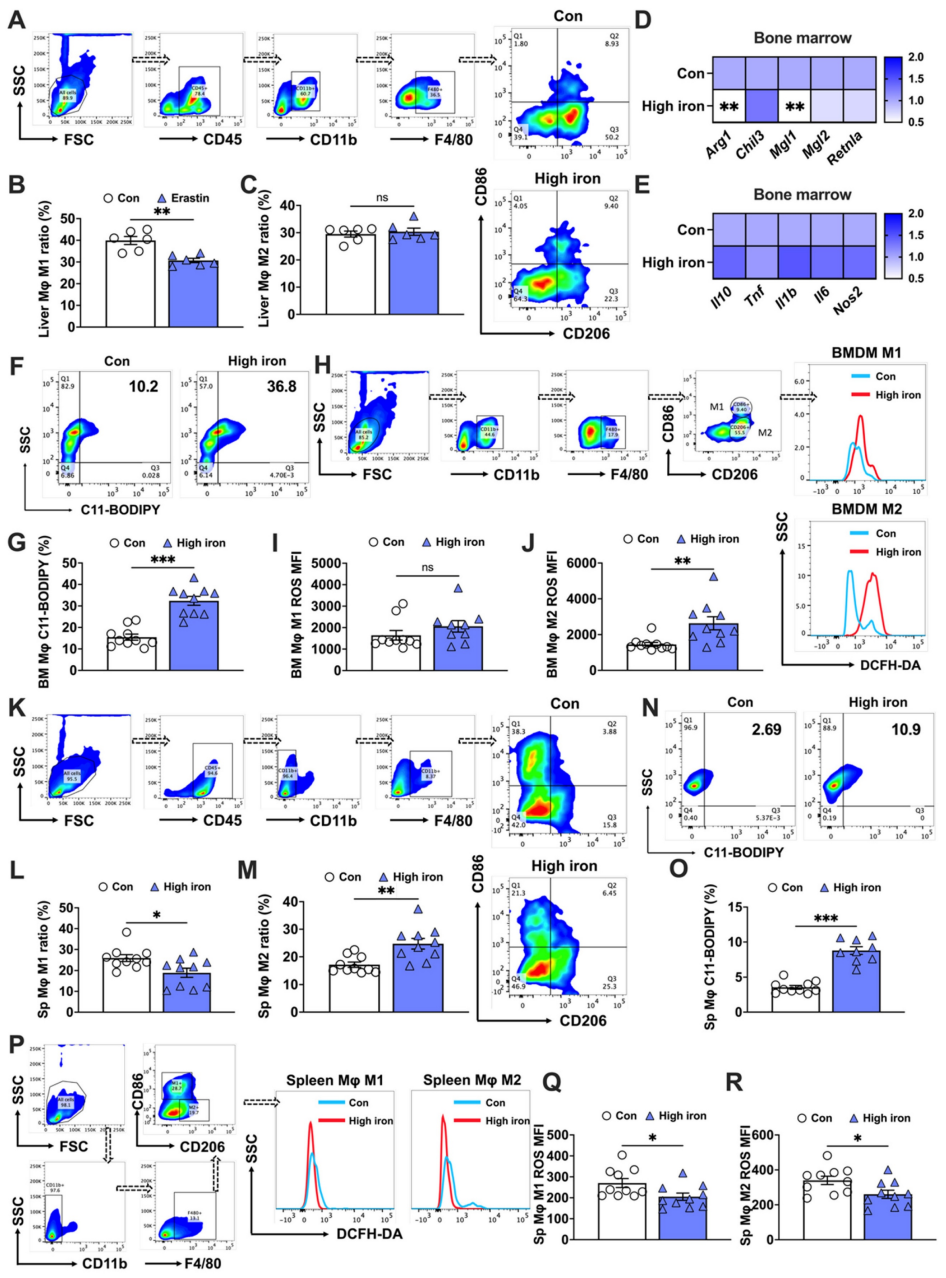
Splenic and bone marrow M1 and M2 macrophages exhibit distinct responses to iron-induced ferroptosis. C57BL/6 J mice were fed a diet containing 5,000 mg/kg of iron for 5 weeks. Post-dissection, femur and tibia bone marrow and spleens were collected. Flow cytometry was used to assess the proportions of M1 and M2 macrophage subtypes and the impact on key indicators of ferroptosis in macrophages derived from different tissue. (A) Flow cytometry gating strategy for bone marrow macrophages in mice; (B) bone marrow M1 macrophage (CD45^+^, CD11b^+^, F4/80^+^, and CD86^+^) ratio (n=10); (C) bone marrow M2 (CD45^+^, CD11b^+^, F4/80^+^, and CD206^+^) macrophage ratio (n=9 or 10); (D) mRNA expression of M2-related markers (*Arg1*, *Chil3*, *Mgl1*, *Mgl2*, and *Retnla*) (n=9 or 10); (E) mRNA expression of M1-related markers (*Il10*, *Tnf*, *Il1b*, *Il6*, and *Nos2*) (n=10); and (F) lipid peroxidation (C11-BODIPY) flow cytometry image; (G) C11-BODIPY positive rate (n=10). (H) Flow cytometry gating strategy for M1 and M2 bone marrow macrophage ROS detection; (I) bone marrow M1 macrophage ROS (CD11b^+^, F4/80^+^, CD86^+^, and DCFH-DA^+^) mean fluorescence intensity (MFI) (n=9 or 10); (I) bone marrow M2 macrophage ROS (CD11b^+^, F4/80^+^, CD206^+^, and DCFH-DA^+^) MFI (n=10); (K) flow cytometry gating strategy for splenic macrophages in mice; (L) splenic M1 macrophage (CD45^+^, CD11b^-^, F4/80^+^, and CD86^+^) ratio (n=10); (M) splenic M2 (CD45^+^, CD11b^-^, F4/80^+^, and CD206^+^) macrophage ratio (n=10); (N) C11-BODIPY flow cytometry image; (O) C11-BODIPY positive rate (n=10). (P) Flow cytometry gating strategy for M1 and M2 splenic macrophage ROS detection; (Q) splenic M1 macrophage ROS (CD11b^-^, F4/80^+^, CD86^+^, and DCFH-DA^+^) MFI (n=10); and (R) splenic M2 macrophage ROS (CD11b^-^, F4/80^+^, CD206^+^, and DCFH-DA^+^) MFI (n=10). ROS = reactive oxygen species; DCFH-DA = 2',7'-dichlorodihydrofluorescein diacetate. Student's t test was used to determine statistical significance, defined as * P < 0.05, **P < 0.01, and ***P < 0.001.

The effects of iron on macrophage polarization remain controversial. Some studies found that iron promoted M2 macrophage polarization, thereby reducing the pro-inflammatory M1 response 6, while other studies indicated that iron accumulation induced M1 polarization and increased inflammatory responses 19. Therefore, further research is needed to better understand the mechanisms and effects of iron on macrophage polarization. In our in vivo studies, mice were subjected to a diet containing 5,000 mg/kg of iron, which helped elucidate the effects of high iron on different macrophage populations. First, we observed a significant reduction in the bone marrow M2 macrophage level, which contrasted with the negligible effect on M1 macrophage levels (Figs. 1A-C). This was substantiated by mRNA analyses showing a decrease in M2-specific markers (*Arg1* and *Mgl1*) (Fig. 1D), while M1 markers (*Il10*, *Tnf*, *Il1b*, *Il6*, and *Nos2*) remained unaffected (Fig. 1E). Additionally, lipid peroxidation, indicative of ferroptosis, was evidenced by a surge in the number of C11-BODIPY^+^ cells under high iron conditions (Figs. 1F and G). The increased iron levels caused a more marked rise in reactive oxygen species (ROS) in bone marrow M2 macrophages than in M1 macrophages (Figs. 1I and J). This suggested a differential susceptibility to ferroptosis under high iron conditions, and highlighted the higher resistance of bone marrow M1 macrophages to ferroptosis.

The splenic macrophage response of mice fed a high iron diet revealed distinct outcomes compared to those of bone marrow macrophages. The elevated iron levels notably increased the M2 macrophage proportion while reducing M1 macrophages (Figs. 1K-M). In contrast to bone marrow macrophages, splenic macrophages exhibited less pronounced lipid peroxidation (C11-BODIPY^+^) in response to high iron conditions, although the cell counts and staining levels were similar (Figs. 1N and O). Additionally, both M1 and M2 splenic macrophages had significant reductions in ROS levels under high iron conditions, with M2 macrophages exhibiting a more marked decrease (Figs. 1P-R). These findings implied that macrophage sensitivity to iron-induced ferroptosis was tissue-specific and polarization-dependent. We hypothesized a sensitivity ranking to iron-induced ferroptosis, with bone marrow M2 macrophages the most susceptible, followed by bone marrow M1, splenic M1, and splenic M2 macrophages.

To investigate tissue-specific ferroptosis sensitivity beyond the spleen and bone marrow, we extended our analysis to liver-resident Kupffer cells and alveolar macrophages using an erastin-induced ferroptosis model. Flow cytometry analysis revealed that erastin treatment significantly reduced the proportion of M1-like kupffer cells in the liver (Sup. Figs. 1A and C), whereas the proportion of M2-like kupffer cells remained unaffected (Sup. Figs. 1A and B). Consistently, RT-PCR analysis showed that erastin markedly suppressed the expression of classical M1 macrophage markers including *Il10*, *Tnf*, *Il1b*, *Il6*, and *Cd86*, while only *Chil3*, an M2-associated marker, was significantly decreased in liver tissue (Sup. Figs. 1D and E). In contrast, the response of alveolar macrophages to erastin exhibited a different trend. The proportion of M1-like alveolar macrophages remained unchanged following erastin exposure (Sup. Figs. 1F and H), while M2-like alveolar macrophages showed a significant increase (Sup. Figs. 1F and G). RT-PCR further demonstrated that erastin selectively upregulated the expression of M1 markers *Il1b* and *Nos2*, and significantly increased M2-associated genes including *Chil3*, *Mgl1*, *Mgl2*, and *Retnla*, consistent with the flow cytometric profiling results (Sup. Figs. 1G and H).Together, these findings highlight a distinct ferroptosis sensitivity pattern between liver and alveolar macrophage subsets, suggesting a tissue-specific regulation of macrophage susceptibility to ferroptotic stress.

### In vitro validation of the different sensitivity to ferroptosis of bone marrow and splenic macrophages

**Figure 2:**
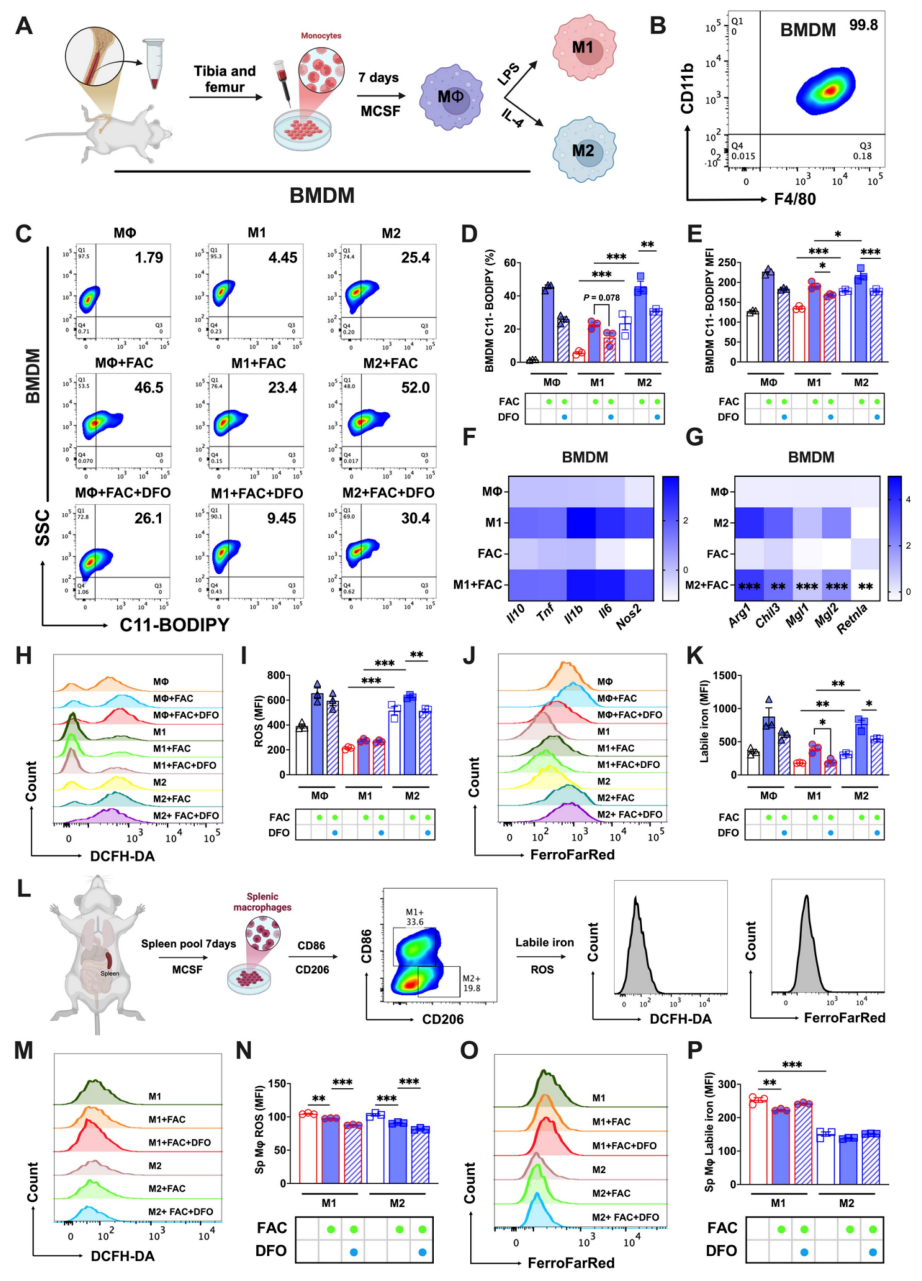
In vitro cultured bone marrow and splenic macrophages replicate the sensitivity differences in iron-induced ferroptosis. We cultured bone marrow-derived macrophages (BMDMs) and splenic macrophages in vitro and examined key indicators of ferroptosis. (A) Flowchart of the isolation, culturing, and polarization of bone marrow macrophages. (B) Identification of bone marrow macrophages as double positive for F4/80 and CD11b; (C) lipid peroxidation (C11-BODIPY) flow cytometry image; (D) C11-BODIPY positive rate (n=3); (E) C11-BODIPY mean fluorescence intensity (MFI) (n=3); (F) detection of M1 BMDM markers (*Il10*, *Tnf*, *Il1b*, *Il6*, and *Nos2*) by RT-PCR (n=4); and (G) detection of M2 BMDM markers (*Arg1*, *Chil3*, *Mgl1*, *Mgl2*, and *Retnla*) by RT-PCR (n=4). (H) DCFH-DA total ROS flow cytometry histogram; (I) total ROS MFI (n=3); (J) FerroFarRed labile iron pool flow cytometry histogram; (K) labile iron MFI (n=3); and (L) splenic macrophage culture and analysis. Briefly, macrophages were cultured in vitro with 20 ng/mL MCSF for 7 d. Post-treatment, they were harvested for flow cytometric analysis. Using CD86 and CD206 markers, M2 and M1 macrophages were distinguished, followed by assessments of total ROS and labile iron levels in differentiated subtypes. (M) DCFH-DA total ROS flow cytometry histogram; (N) total ROS MFI (n=3); (O) FerroFarRed labile iron pool flow cytometry histogram; and (P) labile iron MFI (n=3). LPS = lipopolysaccharide; MCSF = macrophage colony stimulating factor; Sp Mφ = splenic macrophages; ROS = reactive oxygen species; DCFH-DA = 2',7'-dichlorodihydrofluorescein diacetate. One-way ANOVA (Tukey) was used to determine statistical significance, defined as *P < 0.05, **P < 0.01, and ***P < 0.001.

Next, we developed in vitro models for bone marrow and splenic macrophages to further explore this phenomenon. Bone marrow-derived macrophages (BMDMs) were cultured and identified as detailed in Figs. 2A and B. FAC-treated M2 BMDMs had higher lipid peroxidation levels (C11-BODIPY^+^) than those of M1. This peroxidation was mitigated upon treatment with the iron chelator DFO, as shown in Figs. 2C-E. Aligning with our previous findings, FAC significantly decreased the expression of M2 BMDM-specific markers (*Arg1*, *Chil3*, *Mgl1*, *Mgl2*, and *Retnla*), but did not affect M1 markers (*Il10*, *Tnf*, *Il1b*, *Il6*, and *Nos2*) (Figs. 2F and G). Elevated ROS (Figs. 2H and I) and labile iron levels (Figs. 2J and K) were noted in FAC-treated M2 BMDMs compared to M1 BMDMs. Mature splenic macrophages cultured in vitro were identified as M1 or M2 types using CD206 and CD86 markers (using the procedure shown in Fig. 2L). FAC treatment reduced ROS levels in both types, with M2 cells exhibiting lower ROS levels under both treated and untreated conditions (Figs. 2M and N). Labile iron levels in M2 macrophages were also notably lower than in M1 macrophages under both high and normal iron conditions (Figs. 2O and P). Ferroptosis affects cell viability, and in subsequent experiments, we observed that treatment with FAC and the ferroptosis inducer RSL3 produced a higher number of bright-appearing dead cells under optical microscopy (Sup. Figs. 2C and D), and more dead cells were found in M2 macrophages than in M1 macrophages, as indicated by PI staining (Sup. Figs. 2E and F). Additionally, under ferroptosis activation conditions, the number of live M2 macrophages stained with CAM was lower than that of M1 macrophages (Sup. Figs. 2E and F). However, these phenomena were not observed in splenic macrophages under different polarization conditions (Sup. Figs. 2G-J). These findings suggested that in vitro models of bone marrow and splenic macrophages effectively replicated in vivo reactions to iron-induced ferroptosis.

### Different labile iron levels contribute to disparities in the sensitivity of BMDM and splenic macrophages to ferroptosis

**Figure 3:**
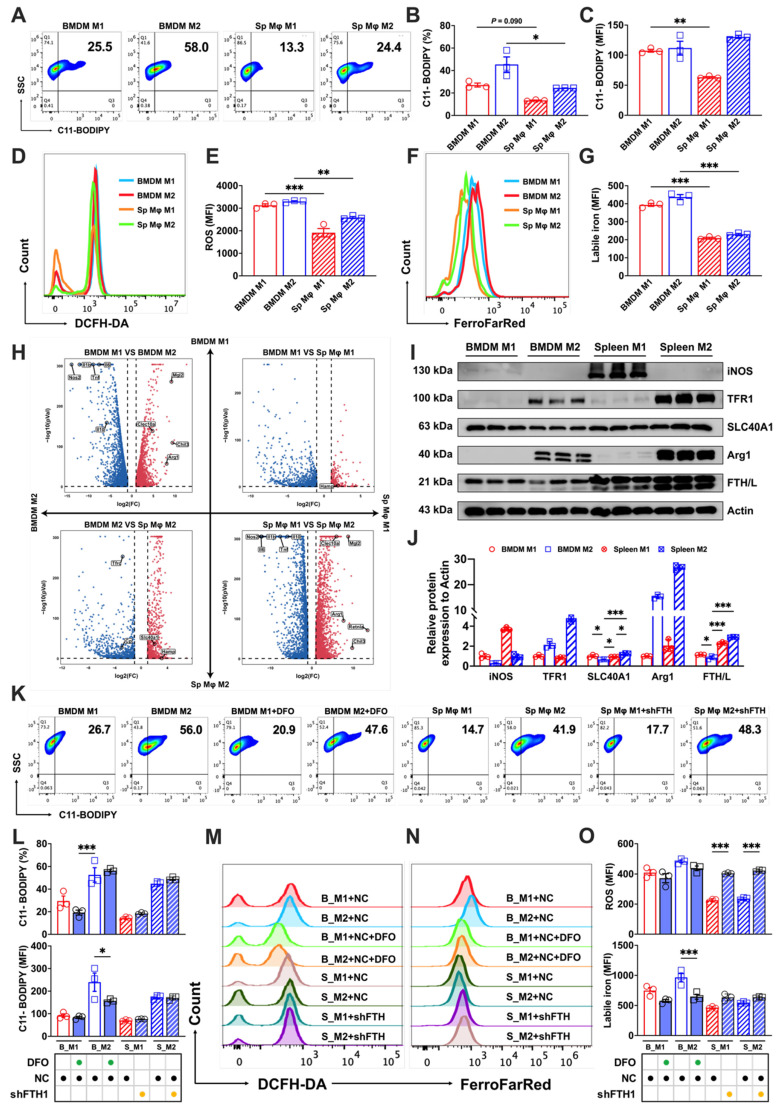
Variations in labile iron levels significantly influence the susceptibility of BMDMs and splenic macrophages to ferroptosis. We pooled spleen and bone marrow cells from three mice, cultured them under identical conditions, and collected the cells at the same time. A portion of the cells in each sample underwent flow cytometry to detect core indicators of ferroptosis, while the other portion was subjected to transcriptomic sequencing. (A) Lipid peroxidation (C11-BODIPY) flow cytometry image; (B) percentage of cells positive for lipid peroxidation (n=3); (C) mean fluorescence intensity (MFI) of lipid peroxidation-positive cells (n=3); (D) DCFH-DA total ROS flow cytometry histogram; (E) MFI of ROS-positive cells (n=3); (F) FerroFarRed labile iron pool flow cytometry histogram; and (G) labile iron MFI (n=3). (H) Transcriptomic volcano plot (fold change > 2), in which the second and fourth quadrants represent the expression of marker genes related to M1 and M2 macrophages. The first and third quadrants show the expression levels of genes related to iron metabolism. (I) Western blot bands of iron metabolism-related proteins (TFR1, SLC40A1, and FTH/L), with iNOS as the marker for M1 macrophages and Arg1 the marker for M2 macrophages. (J) Western blot grayscale analysis of bands for iron metabolism-related proteins (n=3); (K) C11-BODIPY flow cytometry image; (L) percentage of cells positive for lipid peroxidation and MFI (n=3); (M and N) DCFH-DA total ROS and FerroFarRed labile iron pool flow cytometry histogram; and (O) MFI of ROS and labile iron-positive cells (n=3). Sp Mφ = splenic macrophages; ROS = reactive oxygen species; DCFH-DA = 2',7'-dichlorodihydrofluorescein diacetate; TFR1 = transferrin receptor 1; SLC40A1 = solute carrier family 40 member 1; FTH/L = ferritin heavy and light chain; Arg1 = Arginase 1; iNOS = Inducible nitric oxide synthase. One-way ANOVA (Tukey) was used to determine statistical significance, defined as * P < 0.05, ** P < 0.01, and *** P < 0.001.

To further explore the varying levels of ferroptosis resistance among tissue-resident macrophages, we used a splenic macrophages culture technique similar to that used to obtain BMDMs. This method enabled the successful polarization of splenic macrophages, as elaborated in Supp. Figs. 2A and B. A comparative analysis of polarized splenic macrophages and BMDMs using flow cytometry showed lower lipid peroxidation (Figs. 3A-C) and total ROS levels (Figs. 3D and E) in splenic macrophages, coupled with lower labile iron levels than their bone marrow counterparts (Figs. 3F and G).

Transcriptome analysis confirmed the successful polarization of splenic and bone marrow macrophages (marked by high expression of the M1 markers *Il10*, *Tnf*, *Il1b*, *Il6*, and *Nos2* or the M2 markers *Arg1*, *Chil3*, *Clec10a*, *Mgl2*, and *Retnla*), and revealed significant differences in iron metabolism-related gene expression of *Slc40a1*, *Hamp*, and *Tfrc* (fold change > 2) between the two (Fig. 3H). Motivated by the differences in the labile iron pool and transcriptome analyses, we investigated levels of iron metabolism-related proteins, and discovered that BMDMs expressed lower levels of SLC40A1 compared to splenic macrophages. Intriguingly, M2 BMDMs showed even lower expression of the iron storage protein FTH/L than other groups (Figs. 3I and J). To probe whether these iron metabolism protein differences contributed to the distinct ferroptosis sensitivities, we treated a BMDM model with DFO to simulate an increased iron efflux and used a splenic macrophage model with reduced FTH expression (shFTH) to simulate a decreased iron flux. The results revealed that DFO treatment markedly lowered lipid peroxidation (Figs. 3K and L) and labile iron levels (Figs. 3N and O) in M2 BMDM macrophages, while macrophages with reduced FTH showed significantly elevated total intracellular ROS in both M1 and M2 splenic macrophages (Figs. 3M and O). These results suggested that the heightened ferroptosis sensitivity of BMDMs, particularly of M2 macrophages, was attributable to their elevated labile iron levels and reduced FTH/L and SLC40A1 expression.

### Autophagic flux produces different labile iron levels and sensitivity of BMDMs to ferroptosis

**Figure 4:**
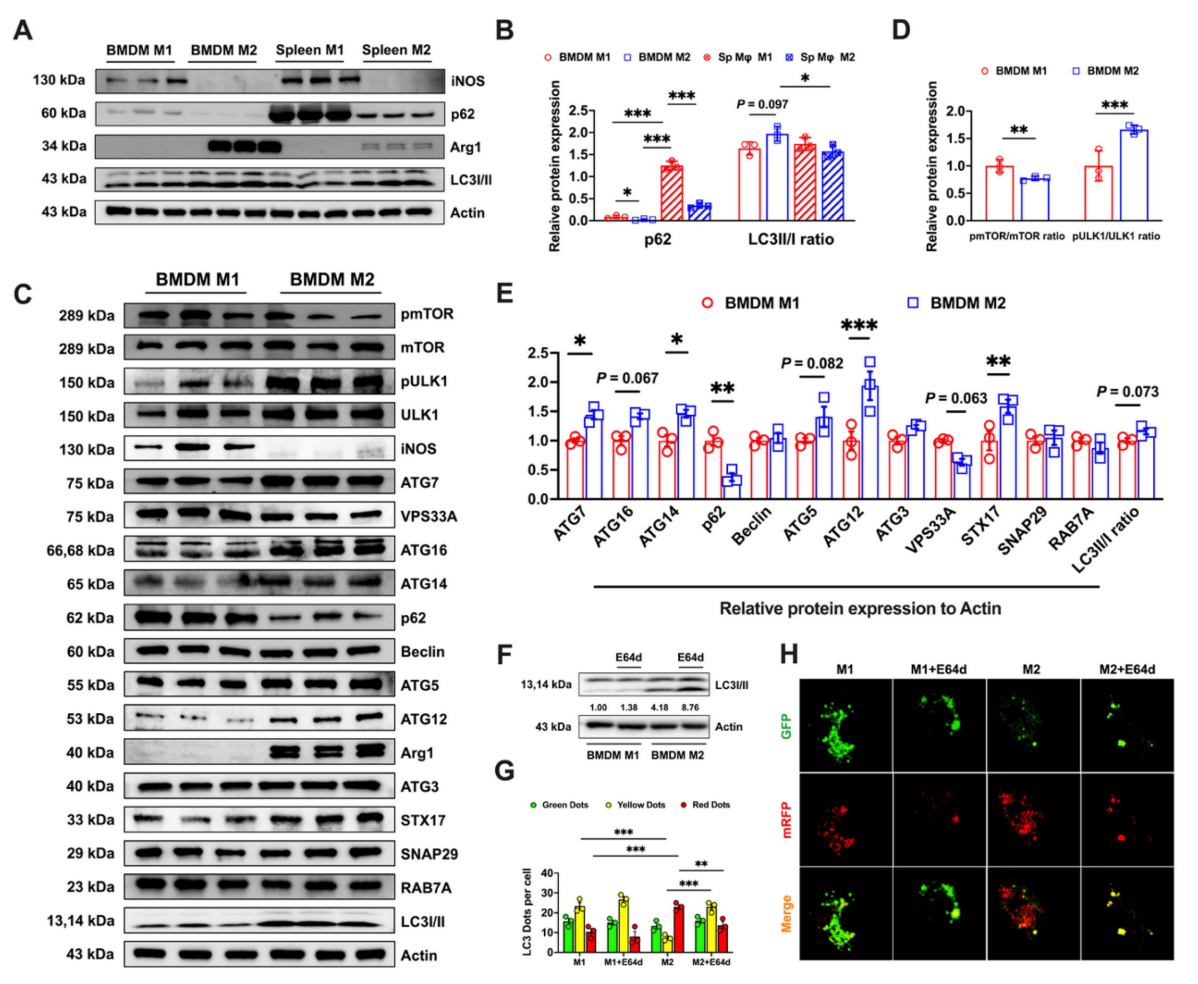
Bone marrow-derived M2 macrophages display elevated expression of autophagy-related proteins and demonstrate a comprehensive autophagic flux. (A) Western blot bands of autophagy flux-related proteins (p62 and ILC3I/II). iNOS is the marker for M1 macrophages and Arg1 is the M2 macrophage marker. (B) Western blot band grayscale analysis of autophagy flux-related proteins (n=3); (C) Western blot bands of autophagy-related proteins (mTOR, pmTOR, ULK1, pULK1, ATG7, VPS33A, ATG16, ATG14, p62, Beclin, ATG5, ATG12, ATG3, STX17, SNAP29, RAB7A, and LC3I/II), with iNOS the marker for M1 macrophages and Arg1 the M2 macrophage marker; (D) Western blot band grayscale analysis for pmTOR/mTOR ratio and pULK1/ULK1 ratio (n=3); and (E) Western blot band grayscale analysis for autophagy-related proteins (n=3). (F) LC3I/II western blot bands and LC3II/I ratio was calculated; (G and H) Monitoring autophagic flux using a dual-labeled adenovirus (mRFP-GFP-LC3); autophagosomes exhibited both GFP and RFP fluorescence signals (yellow dots), while autolysosomes only displayed the RFP fluorescence signal (Red dots). p62 = sequestosome 1; LC3 = microtubule-associated protein light chain 3; mTOR = mammalian target of rapamycin; pmTOR = phosphorylated mammalian target of rapamycin; ULK1 = UNC51-like kinase-1; pULK1 = phosphorylated UNC51-like kinase-1; ATG7 = autophagy-related 7; VPS33A = vacuolar protein sorting 33 homolog A; ATG16 = autophagy-related 16; ATG14 = autophagy-related 14; Beclin = coiled-coil, moesin-like BCL2-interacting protein; ATG5 = autophagy-related 5; ATG12 = autophagy-related 12; ATG3 = autophagy-related 3; STX17 = syntaxin 17; SNAP29 = synaptosome associated protein 29; RAB7A = RAS-related protein Rab-7a; Arg1 = Arginase 1; iNOS = Inducible nitric oxide synthase. One-way ANOVA (Tukey) was used to determine statistical significance, defined as * P < 0.05, ** P < 0.01, and *** P < 0.001.

FTH/L is the primary intracellular iron storage protein, which sequesters excess iron and thereby reducing its availability in the labile pool 20. Ferritin, bound with nuclear receptor coactivator 4 (NCOA4), is targeted for lysosomal degradation in a specialized form of autophagy known as ferritinophagy 11. According to previous research, p62 is as a selective autophagy receptor that recognizes specific cargo for degradation and delivers it to autophagosomes 21. By monitoring LC3-II levels and p62 degradation, the autophagic flux can be assessed 22. When analyzing the autophagic flux in bone marrow and splenic M1 and M2 macrophages, we found that p62 expression followed the order M2 BMDMs < M1 BMDMs < spleen M2 < spleen M1 macrophages, whereas the ratio of LC3I to LC3II protein levels was observed in the order M2 BMDMs > spleen M1 macrophages > M1 BMDMs > spleen M2 macrophages (Figs. 4A and B). These results suggested that autophagic flux was more complete in BMDMs than in splenic macrophages. Given this observation, we further explored the autophagy-related protein expression between M1 and M2 BMDMs. M2 macrophages exhibited a significantly decreased pmTOR/mTOR ratio (Fig. 4C and D) accompanied by increased pULK1/ULK1 ratio (Figure 4C and D), indicating mTORC1 inhibition-triggered ULK1 activation. In addition, the results revealed significantly higher expression of the autophagic structural proteins ATG7, ATG16, ATG14, ATG5, and ATG12 in M2 macrophages. Additionally, M2 cells expressed higher levels of the autophagic fusion protein STX17, although VPS33A expression was notably reduced (Figs. 4C and E). In our findings, M2 BMDM macrophages consistently exhibited lower p62 expression and a higher LC3II/LC3I ratio compared to M1 cells (Figs. 4C and E), suggesting complete autophagic flux in M2 macrophages but potentially stalled late-stage (fusion) autophagy in M1 macrophages. To confirm this hypothesis, we assessed the autophagic flux in BMDMs. Using E64d, we found that LC3II/I ratio remained unchanged in M1 macrophages but increased in M2 macrophages, indicating enhanced autophagic flux in the latter (Fig. 4F). Furthermore, mCherry-GFP-LC3 imaging revealed more yellow puncta (autophagosomes) in M1 and predominant red puncta (autolysosomes) in M2, suggesting more active autophagic flux in M2 cells. Upon E64d treatment, M2 cells showed increased yellow and decreased red puncta, confirming an intact and dynamic autophagic flux (Figs. 4G and H).

Significant differences in the autophagic level and flux among the polarized BMDMs led us to question whether these differences controlled BMDM sensitivity to ferroptosis. By applying autophagy activators (torin1 and rapamycin) and inhibitors (BAFA1 and HCQ) in the experimental procedures detailed in Sup. Fig. 3A, we observed distinct cell morphological responses in polarized BMDMs (Sup. Fig. 3B) specific to the modifiers.

**Figure 5:**
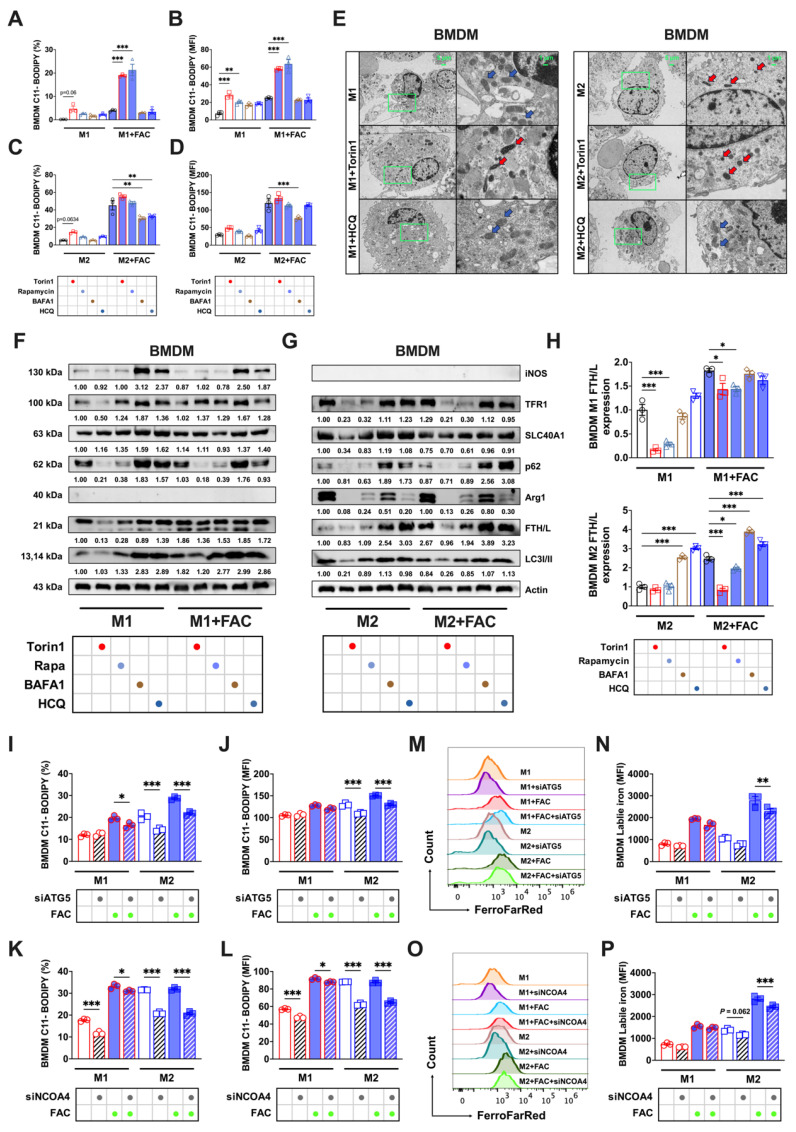
Variability in the BMDM response to ferroptosis controlled by the autophagic flux. We treated BMDMs with autophagy activators (1 μM torin1 or 5 μM rapamycin) and autophagy inhibitors (100 nM BAFA1 or 10 μM HCQ). The specific treatment process is shown in a flowchart in Sup. Fig. 2A. In addition, silencing ATG5 and NCOA4 genes mimic the control of autophagy inhibitors on ferroptosis in both bone marrow-derived and splenic macrophages. (A to D) Percentage of cells positive for lipid peroxidation (C11-BODIPY) in polarized BMDMs and mean fluorescence intensity (MFI) (n=3). (E) BMDM TEM images. Red represents the mitochondrial ferroptosis phenotype, while blue represents normal mitochondria. (F and G) Western blot bands of iron metabolism-related proteins (TFR1, SLC40A1, and FTH/L), and autophagic flux-related proteins (p62 and LC3I/II) in M1 and M2 BMDMs, with iNOS the marker for M1 macrophages and Arg1 the marker for M2 macrophages; and (H) M1 and M2 BMDM gray value quantification of western blot bands for FTH/L (n=3). (I to L) Percentage of BMDMs positive for lipid peroxidation and mean fluorescence intensity (MFI) (n=3); (M and O) BMDM FerroFarRed labile iron pool flow cytometry histogram; and (N and P) BMDM labile iron MFI (n=3). p62 = sequestosome 1; LC3 = microtubule-associated protein light chain 3; TFR1 = transferrin receptor 1; SLC40A1 = solute carrier family 40 member 1; FTH/L = ferritin heavy and light chain; Arg1 = Arginase 1; iNOS = Inducible nitric oxide synthase; ATG5 = autophagy-related 5; NCOA4 = nuclear receptor coactivator 4. One-way ANOVA (Tukey) was used to determine statistical significance, defined as * P < 0.05, ** P < 0.01, and *** P < 0.001.

With FAC treatment, M1 macrophage lipid peroxidation was enhanced by autophagy activators (Figs. 5A and B) whereas this was suppressed in M2 macrophages by autophagy inhibitors (Figs. 5C and D). Ferroptosis often exhibits mitochondrial changes, such as a reduced size and increased membrane density 12. TEM analyses of BMDMs revealed notable changes in the mitochondria of M1 macrophages following treatment with torin1, characterized by shrunken mitochondria structures (red arrows). This trend was also observed in M2 macrophages (red arrows) regardless of whether they were treated with autophagic activators. However, treatment with HCQ restored the mitochondrial morphology to a normal state (Fig 5E, blue arrows). Subsequently, we evaluated the levels of iron metabolism proteins at different autophagic states. Fluctuations in p62 and LC3I/II ratio were indicative of changes in autophagic flux 21, but there were no notable alterations in the iron metabolism proteins (TFR1, SLC40A1, or FTH/L) in M1 BMDMs, as shown in Figs. 5F-H. In contrast, M2 BMDMs showed significantly decreased FTH/L and TFR1 protein levels when treated with autophagy activators but increased levels when treated with inhibitors (Figs. 5F-H). To verify how the autophagic flux influenced the labile iron levels, we found that inhibiting autophagy markedly reduced Fe^2+^ in bone marrow M2 macrophages (Sup. Figs. 4A and B). These results support our hypothesis that M2 BMDM macrophages, due to increased and complete autophagy, experienced FTH/L degradation, leading to high labile iron levels and reduced resistance to ferroptosis.

To specifically investigate whether NCOA4-mediated ferritinophagy contributed to the increased susceptibility of M2 BMDM macrophages to iron-induced ferroptosis, we conducted knockdown experiments of ATG5 (a crucial protein in autophagosome formation) and NCOA4 23. Initially, we verified the knockdown efficacy of ATG5 and NCOA4 siRNA through FAM fluorescence and Western blot analyses, which confirmed efficient transfection (Sup. Figs. 5A and D) and knockdown (using siRNA ATG5-1, Sup. Figs. 5B and E, and siRNA NCOA4-1, Sup. Figs. 5C and F). The results showed that ATG5 and NCOA4 knockdown significantly reduced lipid peroxidation (Figs. 5I-J) and Fe^2+^ levels (Figs. 5M-N) in M2 BMDMs with and without FAC treatment. However, in M1 BMDMs, there was a less pronounced effect on lipid peroxidation (Figs. 5K-L) and labile iron levels (Figs. 5O-P).

### Autophagy modulation and ferroptosis sensitivity in splenic macrophages

**Figure 6:**
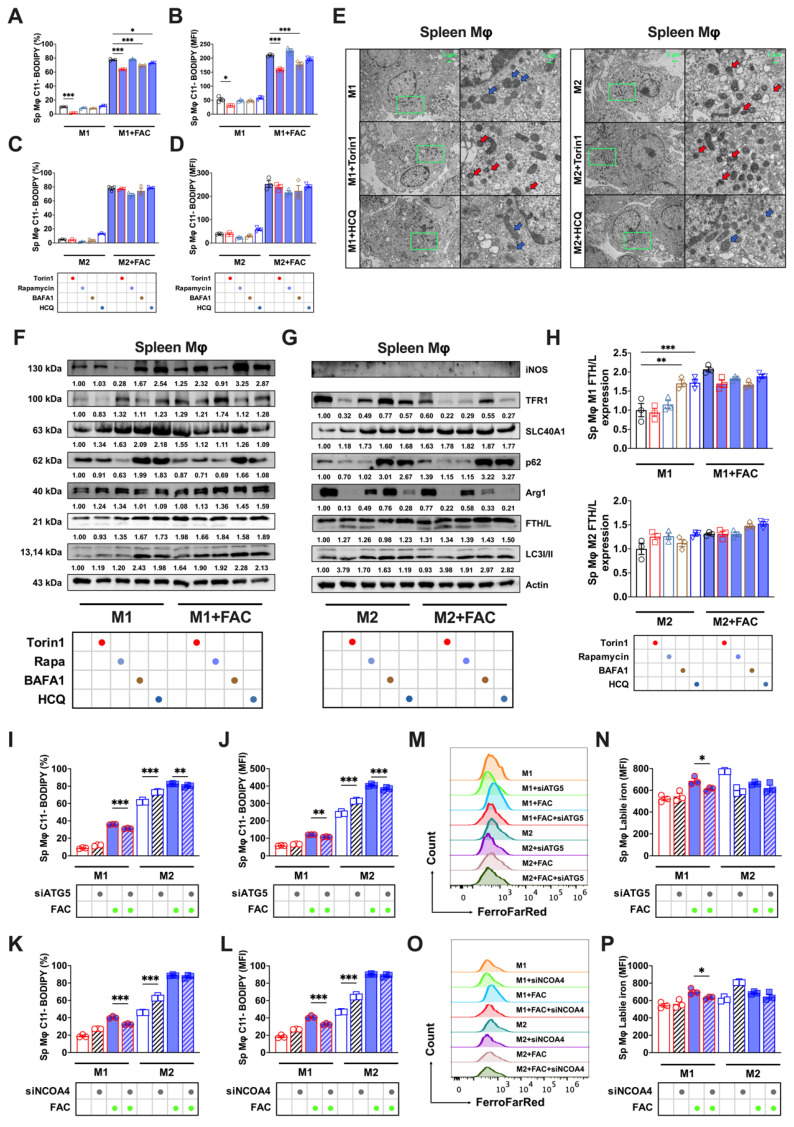
Autophagic flux did not produce ferroptosis sensitivity in splenic macrophages. We treated splenic macrophages with autophagy activators (1 μM torin1 or 5 μM rapamycin) and autophagy inhibitors (100 nM BAFA1 or 10 μM HCQ). The specific treatment process is shown in flowchart of Sup. Fig. 2A. In addition, silencing ATG5 and NCOA4 genes mimic the control of autophagy inhibitors on ferroptosis in both bone marrow-derived and splenic macrophages. (A to D) Percentage of the cell positive rates and mean fluorescence intensity (MFI) of lipid peroxidation (C11-BODIPY) in polarized splenic macrophages (n=3). (E) Splenic macrophage TEM images; red represents the mitochondrial ferroptosis phenotype, and blue represents normal mitochondria. (F and G) Western blot bands of iron metabolism-related proteins (TFR1, SLC40A1, and FTH/L) and autophagic flux-related proteins (p62 and LC3I/II) in splenic M1 and M2 macrophage, with iNOS the marker for M1 macrophages and Arg1 the marker for M2 macrophages; and (H) Splenic M1 and M2 macrophage gray value quantification of Western blot bands for FTH/L (n=3). (I to L) Percentage of Splenic macrophage positive for lipid peroxidation and mean fluorescence intensity (MFI) (n=3); (M and O) Splenic macrophage FerroFarRed labile iron pool flow cytometry histogram; and (N and P) Splenic macrophage labile iron MFI (n=3). Sp Mφ and spleen Mφ = splenic macrophages; p62 = sequestosome 1; LC3 = microtubule-associated protein light chain 3; TFR1 = transferrin receptor 1; SLC40A1 = solute carrier family 40 member 1; FTH/L = ferritin heavy and light chain; Arg1 = Arginase 1; iNOS = Inducible nitric oxide synthase; ATG5 = autophagy-related 5; NCOA4 = nuclear receptor coactivator 4. One-way ANOVA (Tukey) was used to determine statistical significance, defined as * P < 0.05, ** P < 0.01, and *** P < 0.001.

Splenic macrophages, characterized by higher FTH/L and SLC40A1 expression and thus lower labile iron levels, demonstrated greater ferroptosis resistance to BMDMs. Autophagy modulation of splenic macrophages (cell morphology detailed in Sup. Fig. 3C) did not yield lipid peroxidation changes analogous to those in BMDMs. Autophagy inhibition significantly reduced lipid peroxidation only in high iron-treated M1 cells (Figs. 6A, and B), with no marked effect on M2 cells (Figs. 6C and D). In addition, autophagic regulation did not influence the M1 or M2 total ROS levels in either FAC-treated or non-treated splenic macrophages (Sup. Figs. 4C-F). The TEM results showed that under the influence of autophagic flux regulators, spleen M1 and M2 macrophages did not exhibit changes as pronounced as those observed in bone marrow macrophages (Fig. 6E). Western blot analyses revealed that enhancing or inhibiting autophagic flux had no significant effect on FTH/L, TFR1, or SLC40A1 in M1 macrophages. However, under enhanced autophagic flux conditions, a significant reduction in TFR1 protein expression was observed in M2 macrophages (Figs. 6F-H). These observations suggested a different sensitivity of FTH/L to autophagy modulation between BMDMs and splenic macrophages, potentially underpinning the differences in ferroptosis sensitivity.

**Figure 7:**
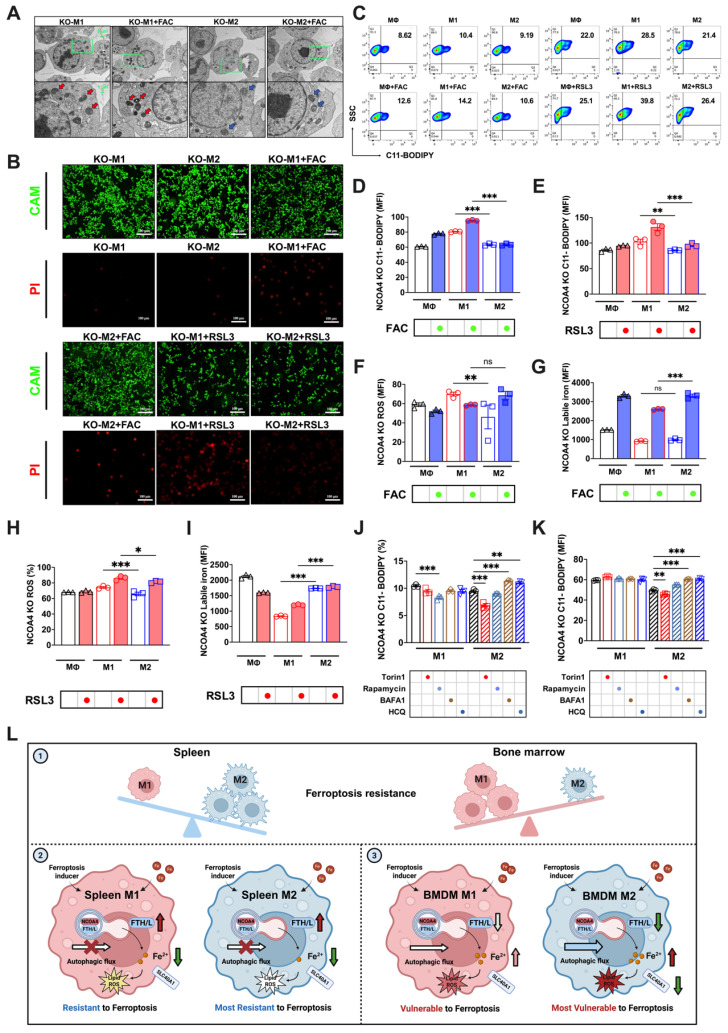
Knocking out NCOA4 reverses the susceptibility of M1 and M2 macrophages to FAC and RSL3-induced ferroptosis. (A) NCOA4-KO RAW264.7 cell TEM images, in which red represents the mitochondrial ferroptosis phenotype, and blue represents normal mitochondria; (B) Cell viability staining. CAM green signal indicates live cells, while PI red signal denotes dead cells. (C) NCOA4-KO RAW264.7 cell lipid peroxidation (C11-BODIPY) flow cytometry image; (D and E) percentage of NCOA4-KO RAW264.7 cells positive for lipid peroxidation (C11-BODIPY) and mean fluorescence intensity (MFI) (n=3); (F to G) NCOA4-KO RAW264.7 cell DCFH-DA total ROS and labile iron flow cytometry histogram; and MFI of ROS-positive and labile iron-positive NCOA4-KO polarized RAW264.7 macrophages treated with FAC (n=3). (H and I) NCOA4-KO RAW264.7 cell MFI of ROS-positive and labile iron-positive NCOA4-KO polarized RAW264.7 cells treated with RSL3 (n=3); and (J and K) NCOA4-KO polarized RAW264.7 cell MFI percentage of C11-BODIPY (n=3); (L) Mechanism diagram. By investigating intracellular iron regulation, we elucidated the means by which ferritinophagy modulates the susceptibility to ferroptosis of differently polarized macrophages in the spleen and bone marrow. The main experimental results are as follows. (1) In vivo analyses revealed a distinct resistance profile against high iron-induced ferroptosis among macrophage subtypes, with bone marrow M2 macrophages the most susceptible, followed by bone marrow M1, splenic M1, and the splenic M2 macrophages. (2) Due to a relatively impaired autophagic flux (red cross on autophagic flow arrow), enhanced expression of SLC40A1 and FTH/L (dark red arrow) in splenic macrophages correlates with reduced labile iron levels (dark green arrow), thereby conferring increased resistance to ferroptosis. (3) Bone marrow M2 macrophages, due to their high autophagy levels and complete autophagic flow (thick blue arrow), undergo ferritinophagy-mediated degradation of FTH/L (darker green arrow), leading to an increased labile iron content (darker red arrow), rendering these macrophages the least resistant to ferroptosis. ROS = reactive oxygen species; DCFH-DA = 2',7'-dichlorodihydrofluorescein diacetate; NCOA4 = nuclear receptor coactivator 4; SLC40A1 = solute carrier family 40 member 1; FTH/L = ferritin heavy and light chain; CAM = cell adhesion molecule; PI = propidium iodide. One-way ANOVA (Tukey) was used to determine statistical significance, defined as * P < 0.05, ** P < 0.01, and *** P < 0.001.

Previous results indicated that modulating the autophagic flux had no significant impact on ferroptosis in splenic macrophages. To further validate this finding, we knocked down ATG5 and NCOA4 in both splenic macrophage models. First, we observed a notable decrease in lipid peroxidation (Figs. 6I-J) and labile iron pools (Figs. 7M-N) in M1 macrophages, with a limited impact on M2 splenic macrophages derived using the procedures shown in Sup. Figs. 2A and B. Second, using a gating strategy based on CD206 and CD86 markers, we similarly observed that modulating the autophagic flux did not significantly affect the total ROS levels in splenic M1 and M2 macrophages (Sup. Figs. 5G and H). This experiment further confirmed that ferritinophagy in M2 BMDM macrophages targeting FTH/L degradation led to the accumulation of lipid peroxidation and Fe^2+^. In contrast, M1 BMDM macrophages and splenic macrophages showed less pronounced effects on lipid peroxidation and labile iron levels.

### NCOA4 knockout reverses the sensitivity of M1 and M2 macrophages to ferroptosis

Given the possible role of ferritinophagy in determining the sensitivity of BMDMs to ferroptosis, we attempted to knock out a key participating gene, NCOA4 (knockout validation shown in Sup. Fig. 7A), thereby disrupting the autophagy-controlled iron metabolism imbalance, to further investigate the differences in ferroptosis sensitivity between M1 and M2 macrophages. Considering the high similarity between polarized RAW264.7 and BMDM responses in iron-induced ferroptosis (FAC-induced peroxidation responses shown in Sup. Figs. 6A-C and cell viability shown in Sup. Fig. 6D), we developed a RAW264.7 *NCOA4* knockout strain for our experiments. Our observations revealed that *NCOA4* knockout in both M1 and M2 macrophages under FAC treatment did not produce significant cell morphological differences (Sup. Fig. 7A).

However, when treated with the ferroptosis inducer RSL3, more M1 than M2 floating dead cells were observed (Sup. Fig. 7A). Additionally, cell viability staining indicated more M1 than M2 PI-positive dead cells with both FAC and RSL3 treatments (Fig. 7B). The TEM results were consistent with these findings, and showed varying degrees of mitochondrial darkening, shrinkage, and morphological damage in M1 macrophages (red arrows) regardless of FAC treatment, while M2 macrophages exhibited notably better mitochondrial morphology (blue arrows) (Fig. 7A). We also assessed typical ferroptosis indicators, including lipid peroxidation and total ROS. With both FAC and RSL3 treatment, M1 macrophages exhibited significantly higher lipid peroxidation (Figs. 7C-E) and total ROS levels (Figs. 7F and H) than M2 macrophages. Moreover, autophagy inhibitors failed to reduce lipid peroxidation in M2 macrophages with *NCOA4* knocked out (Figs. 7J and K). Finally, we measured changes in the labile iron levels after disrupting ferritinophagy. The results showed no significant difference in labile iron levels between M1 and M2 macrophages; however, with FAC treatment, labile iron significantly accumulated in M2 but not M1 macrophages (Fig. 7G). This phenomenon was also observed in M1 and M2 polarized knockout cells treated with RSL3 (Fig. 7I). In summary, upon knocking out the key ferritinophagy protein, the sensitivity of M1 and M2 macrophages to ferroptosis reversed, with M1 macrophages exhibiting poorer resistance.

## Discussion

### Tissue-specific ferroptosis sensitivity in macrophages

Recent advancements in technology, including innovative mouse models, spatial single-cell sequencing, and analysis pipelines, have significantly enhanced our comprehension of macrophage development and function across diverse tissues over the past decade 24. It is becoming increasingly evident that tissue-resident macrophages are not merely passive responders to stimuli or infections. Instead, they occupy a critical position at the junction of tissue homeostasis and pathogenesis where they can contribute to or even initiate diseases if their fundamental homeostatic functions are disrupted 25. Despite our expanding knowledge of macrophage responses within their distinct niches, a significant gap remains in our understanding of how different macrophage populations within the body integrate and react to identical signals.

Ferroptosis, a novel form of regulated cell death, is characterized by significant iron accumulation and lipid peroxidation. Recent studies demonstrated a strong association between ferroptosis and the progression of various diseases, particularly cancers 12, 26. Dietary iron overload is a conventional means of inducing ferroptosis 27. Our prior research demonstrated that a dose of 5,000 mg/kg iron induced hepatic ferroptosis 28. Thus, in this experiment, we used the same dietary regimen to observe the response of macrophages to ferroptosis in different tissue compartments and polarization states. In vivo experiments revealed a distinct sensitivity ranking for iron-induced ferroptosis among macrophage populations in the following order (high to low): bone marrow M2, bone marrow M1, splenic M1, and splenic M2 macrophages.

Our extended analysis incorporating liver kupffer cells and alveolar macrophages revealed distinct tissue-specific patterns: erastin treatment preferentially eliminated M1-like kupffer cells while sparing M2 populations, whereas alveolar macrophages exhibited an inverse sensitivity profile. These findings reinforce the concept of microenvironment-driven regulation of ferroptosis susceptibility across tissue compartments.

### *In vitro* validation and technical considerations

To further validate the in vivo findings, we used a well-established polarization induction model to obtain mature BMDMs 15. However, although established cultivation protocols exist for splenic macrophages 16, 17, published studies have not used cytokines to induce differentiation. Therefore, we initially used CD206 and CD86 as markers to distinguish between splenic M1 and M2 macrophages. Due to the strong PE signal from C11-BODIPY and limitations in our detection channels, we restricted our analysis to measuring ROS and labile iron levels. Consistent with our expectations, the differences in ferroptosis sensitivity between bone marrow and splenic macrophages were recapitulated in the in vitro model. However, in future research, using FACS to isolate splenic M1 and M2 macrophages may provide a more effective model. These results suggested that different tissue-resident macrophages may differ in their resistance to ferroptosis, potentially due to heterogeneity and metabolism differences.

### Iron metabolism and ferritinophagy in ferroptosis

To further investigate the reasons behind the differing sensitivities, we reexamined the primary pathways involved in ferroptosis. NRF2-centered and GSH/GPX4 anti-ferroptotic signaling were found to be irrelevant (data not shown). Moreover, transcriptomic analysis revealed no significant changes in the lipid metabolism-associated genes ACSL4 and LOXs (data not shown). Thus, our study primarily shifted focus toward the pathways of iron metabolism and ferritinophagy. The interplay between autophagy and ferroptosis was described previously. First, autophagy deficiency, demonstrated using *Becn1* and *Map1lc3b* knockout cells, led to reduced intracellular iron and diminished lipid peroxidation, resulting in cell survival during erastin-induced ferroptosis 29, 30. Second, erastin-triggered ROS stimulated ROS-induced autophagy and acted as a key regulator of ferritin degradation and TFR1 expression during ferroptosis 30. In this study, we observed that M2 BMDM macrophages, highly susceptible to ferroptosis, had markedly low FTH/L expression. This observation prompted an exploration of ferritinophagy in M2 BMDM macrophages, which revealed a significantly higher autophagy level with complete autophagic flux. Mechanistically, M2 macrophages displayed mTORC1 inhibition and ULK1 activation, driving autophagy-related protein upregulation. Complementary studies identified NRF2 nuclear translocation in M2 cells, potentially coordinating antioxidant responses with ferritinophagy. Upon treating ATG5 and NCOA4 knockdown and NCOA4 knockout RAW264.7 cells with autophagic flux modifiers, we discovered that the ferroptosis sensitivity of M2 BMDMs was regulated by ferritinophagy. However, in splenic macrophages, unlike in BMDMs, we found that autophagic flux regulation and autophagy protein knockdown did not produce significant changes. Notably, TFR1-mediated iron uptake and HO-1-driven heme degradation exhibited tissue-specific contributions, with splenic M1 resistance persisting despite HO-1 elevation, highlighting compartmentalized iron handling strategies. This may be related to the primary role of splenic macrophages in recycling aged red blood cells 31, which involves exposure to large amounts of labile iron released by HO-1 degradation 32. Their robust iron metabolism capacity may endow them with a strong resistance to ferroptosis.

On the other hand, previous research has highlighted the use of iNOS and NO• for determining the resistance of M1 macrophages and microglia to ferroptosis 10. Our study further revealed that the expression of iNOS was regulated by autophagy inhibitors, leading to significant protein accumulation in both M1 BMDM and splenic M1 macrophages (Figs. 5E and K). This observation aligns with reports showing iNOS interaction with the autophagy receptor p62 and its degradation via autophagy in M1 macrophages 33. However, marginal changes in lipid peroxidation levels suggest that the inherently low ferroptosis susceptibility of M1 macrophages may dilute iNOS-mediated protective effects. These findings collectively indicate that iNOS contributes to, but does not exclusively dictate, the ferroptosis resistance profile of macrophage subsets.

Further confirm my findings, single cell analyses of human bone marrow and spleen demonstrated enhanced iron storage capacity in splenic macrophages, with M1 populations showing substantially higher *FTH1* and *FTL* expression than bone marrow counterparts. Pathological models of hematological malignancies revealed disease-specific remodeling. Tumor-associated M2 macrophages exhibited *FTH1* upregulation with concurrent *NCOA4* suppression, suggesting adaptive iron sequestration as a ferroptosis resistance mechanism (Figs. S8I to N).

### Developmental origins and ferroptosis susceptibility

The metabolic and functional differences among macrophages in various tissues may lead to disparities in their sensitivity to ferroptosis. Our hypothesis suggests that the underlying cause of this phenomenon is associated with the diverse differentiation and developmental origins of macrophages in various tissues. During embryogenesis, the fetal liver becomes the primary source of hematopoiesis, giving rise to diverse cell lineages 25. Monocytes originating from the fetal liver colonize most tissues (spleen and liver), excluding the brain. These monocytes contribute to the formation of tissue-resident macrophages, potentially replacing those derived from the yolk sac. After birth, hematopoiesis shifts to the bone marrow, where blood monocyte precursors continuously replenish resident macrophages 34-36. Previous studies revealed that microglia, a specialized type of macrophage located within the brain and spinal cord, share ferroptosis sensitivity characteristics similar to those of bone marrow macrophages 10. However, research has also shown that the behavior of hepatic macrophages mirrors the ferroptosis resistance pattern observed in splenic macrophages. Increased iron levels enhanced the expression of M2 markers in the liver, whereas a deficiency in iron resulted in decreased *Arg1* expression 37. These observations were consistent with our hypothesis that macrophages originating from the fetal liver had different ferroptosis patterns than macrophages originating from the yolk sac and bone marrow. This heterogeneity stems from developmental ontogeny, with yolk sac-derived macrophages exhibiting distinct iron regulation compared to bone marrow-derived populations. Single cell lineage tracing confirmed enhanced iron-recycling signatures (higher expression of *FTH* and *FTL*) in splenic macrophages, aligning with their specialized erythrocyte clearance function. Such developmental programming may underlie their intrinsic resistance to ferroptotic stress.

### Therapeutic implications of targeting ferroptosis in macrophages

Overall, this study significantly advanced our understanding of macrophage heterogeneity and their resistance to ferroptosis in different tissues and identified promising pathways for macrophage regulation. Macrophage regulation stands at the forefront of medical innovation by offering transformative applications across a spectrum of health challenges. In disease treatment, particularly cancer, modulating macrophage activity can significantly enhance the efficacy of immunotherapies, turning the tide in battles against malignancies 38. In the realm of chronic inflammatory conditions, such as rheumatoid arthritis and psoriasis 39, 40, precisely regulated macrophages may provide a means of mitigating inflammation and alleviating suffering. Furthermore, in tissue repair and regeneration, the strategic manipulation of macrophage activity has been shown to accelerate wound healing, making it a vital tool for post-surgical recovery and treatment of chronic wounds 2. Single cell RNA sequencing data from GEO suggests that tumor-associated M2 macrophages exhibit a ferroptosis-resistant phenotype, particularly in AML and SMZL. The observed alterations in iron regulation, including increased *FTH1* and decreased *NCOA4*, point to a shift in iron storage capacity that may contribute to immunosuppression in the tumor microenvironment. Based on these findings, we propose that targeting ferritinophagy to sensitize M2 macrophages to ferroptosis could be a promising strategy to modulate the immune response and enhance anti-tumor immunity. This approach may lead to novel therapeutic avenues for cancer treatment, where reprogramming macrophages could restore immune function and improve treatment outcomes.

### Future perspectives

The tissue-specific differences in ferroptosis sensitivity observed in our study suggest that macrophages in different organs may have distinct regulatory mechanisms governing their susceptibility to ferroptosis. Further research is needed to explore how tissue-specific factors in the liver, lungs, and other organs modulate ferroptosis sensitivity in macrophages, particularly in the context of disease states. Mechanistically, there is a significant need to deepen our understanding of how lipid metabolism and post-transcriptional regulation contribute to ferroptosis sensitivity. The interplay between lipid metabolism and iron homeostasis, as observed in M2 macrophages 41, suggests that lipid peroxidation may synergize with iron accumulation to drive ferroptosis. Additionally, post-transcriptional mechanisms, including the regulation of iron-related genes via iron-responsive elements and post-translational modifications like phosphorylation and ubiquitination, represent another layer of regulation that requires further investigation 42. By exploring these two pathways, we can gain new insights into the complex mechanisms that influence ferroptosis sensitivity, which may uncover novel therapeutic targets for diseases associated with ferroptosis and macrophage dysfunction.

## Conclusion

This study provides critical insights into the tissue-specific resistance of macrophages to high iron-induced ferroptosis. As shown in Fig.7L, in vivo analyses revealed that bone marrow M2 macrophages are the most susceptible to ferroptosis, followed by bone marrow M1, splenic M1, and splenic M2 macrophages. Our findings demonstrate that the resistance to ferroptosis among these macrophage subtypes is closely linked to their iron metabolism and autophagic flux. In splenic macrophages, impaired autophagic flux (red cross on the autophagic flux arrow) and enhanced expression of iron regulators such as SLC40A1 and FTH/L contribute to reduced labile iron levels (green arrow), thus increasing resistance to ferroptosis (the fainter color of the lipid ROS). In contrast, bone marrow M2 macrophages, characterized by high autophagic flux (A thicker autophagic flux arrow) and ferritinophagy-mediated degradation of FTH/L (green arrow), accumulate more labile iron (red arrow), rendering them more prone to ferroptosis (the darker color of the lipid ROS). These findings highlight the complex interplay between iron metabolism, autophagy, and ferroptosis resistance in macrophages, underscoring the importance of tissue-specific factors in shaping immune responses under iron overload conditions.

## Supplementary Material

Supplementary figures and tables, methods.

## Abbreviations

Arg1: arginase 1
ATG14: autophagy-related 14
ATG16: autophagy-related 16
ATG3: autophagy-related 3
ATG5: autophagy-related 5
ATG7: autophagy-related 7
AML_Ctrl: healthy human bone marrow sample
AML: acute myeloid leukemia bone marrow sample
BAFA1: bafilomycin a1
Beclin: coiled-coil, moesin-like bcl2-interacting protein
BMDM: bone marrow-derived macrophage
BSA: bovine serum albumin
CAM: cell adhesion molecule
Cd86: cluster of differentiation 86
DFO: deferoxamine
FAC: ferric ammonium citrate
Retnla: found in inflammatory zone 1
SLC40A1: solute carrier family 40 member 1
FTH/L: ferritin heavy and light chain
Fth1: ferritin heavy chain 1
Ftl1: ferritin light chain
Il10: interleukin 10r
Il1b: interleukin 1β
Il6: interleukin 6
IL-4: interleukin 4
HCQ: hydroxychloroquine
iNOS: inducible nitric oxide synthase
Keap1: kelch-like ech-associated protein 1
LPS: lipopolysaccharide
LC3: microtubule-associated protein light chain 3
mTOR: mammalian target of rapamycin
MCSF: macrophage colony-stimulating factor
Mgl1: macrophage galactose n-acetyl-galactosamine-specific lectin 1
Mgl2: macrophage galactose n-acetyl-galactosamine-specific lectin 2
Mrc1: mannose receptor c-type 1
NCOA4: nuclear receptor coactivator 4
Nos2: nitric oxide synthase 2
NC: negative control
RAB7A: ras-related protein rab-7a
ROS: reactive oxygen species
RSL3: ras-selective lethal 3
SNAP29: synaptosome associated protein 29
p62: sequestosome 1
pmTOR: phosphorylated mammalian target of rapamycin
pULK1: phosphorylated unc51-like kinase-1
PI: propidium iodide
STX17: syntaxin 17
SMZL_Ctrl: healthy human spleen sample
SMZL: splenic marginal zone lymphoma sample
TFR1: transferrin receptor 1
Tnf: tumor necrosis factor α
TEM: transmission electron microscopy
ULK1: unc51-like kinase-1
VPS33A: vacuolar protein sorting 33 homolog a
Chil3: chitinase-like protein 3
