# Supplementary material for: Ferritinophagy activation states determine the susceptibility to ferroptosis of macrophages in bone marrow and spleen
Source: Int J Biol Sci. 2025 Jul 11;21(10):4567–85. doi: 10.7150/ijbs.114545 (PMC12320229; doi:10.7150/ijbs.114545)
Supplement: Supplementary file 1 — Supplementary figures and tables, methods. [file ijbsv21p4567s1.pdf]

**Supplementary materials**

**Sup. Table S1.** Western blot and flow cytometry antibodies.

| Chemicals and reagents | Source | Catalog # |
|---|---|---|
| Ferrous sulfate (FeSO4) | Sigma-Aldrich, St. Louis, MO, United States | 7782-63-0 |
| Ferric ammonium citrate (FAC) | Sigma-Aldrich, St. Louis, MO, United States | F5879 |
| Deferoxamine (DFO) | Sigma-Aldrich, St. Louis, MO, United States | 138-14-7 |
| RSL3 | Selleck, Shanghai, China | S8155 |
| Torin1 | Selleck, Shanghai, China | S2827 |
| Rapamycin | Selleck, Shanghai, China | S1039 |
| Bafilomycin A1 (BAFA1) | Selleck, Shanghai, China | S1413 |
| Hydroxychloroquine sulfate (HCQ) | Selleck, Shanghai, China | S4430 |

**Sup. Table S2.** Primers used in the study.

| Gene | Forward 5’-3’ | Reverse 5’-3’ |
|---|---|---|
| *Arg1* | AGCACTGAGGAAAGCTGGTC | CAGACCGTGGGTTCTTCACA |
| *Mgl1* | TGCAACAGCTGAGGAAGGACTTGA | AACCAATAGCAGCTGCCTTCATGC |
| *Mgl2* | GCATGAAGGCAGCTGCTATTGGTT | TAGGCCCATCCAGCTAAGCACATT |
| *Retnla* | TCCAGCTGATGGTCCCAGTGAATA | ACAAGCACACCCAGTAGCAGTCAT |
| *Chil3* | AGAAGGGAGTTTCAAACCT | GTCTTGCTCATGTGTGTAAGTGA |
| *Cd86* | CTTACGGAAGCACCCCAGAT | TGTAAATGGGCACGGCAGAT |
| *Il6* | AAAATTTCCTCTGGTCTTCTGGAGT | TTCTGTGACTCCAGCTTATCTCTTG |
| *Il1b* | GCTTCCTTGTGCAAGTGTCTGA | TCAAAAGGTGGCATTTCACAGT |
| *Il10* | CCACTCACCTGCTGCTACTCA | TGGTGATCCTCTTGTAGCTCTCC |
| *Nos2* | CAGGTCTTTGACGCTCGGAA | GCCTGAAGTCATGTTTGCCG |
| *Tnf* | TCTCATGCACCACCATCAAGGACT | ACCACTCTCCCTTTGCAGAACTCA |
| *Mrc1* | GTCAGAACAGACTGCGTGGA | AGGGATCGCCTGTTTTCAG |
| *Actb* | TTGTGATGGACTCCGGAGAC | TGATGTCACGCACGATTTCC |

**Sup. Table S3.** Western blot and flow cytometry antibodies.

| **Antibody** | **Source** | **Catalog #** |
|---|---|---|
| Purified rat anti-mouse CD16/CD32 | BD Pharmingen$^{TM}$ | 553141 |
| FITC rat anti-mouse CD45 | BD Pharmingen$^{TM}$ | 553080 |
| Alexa Fluor® 647 rat anti-mouse F4/80 | BD Pharmingen$^{TM}$ | 565853 |
| PerCP-CyTM5.5 rat anti-CD11b | BD Pharmingen$^{TM}$ | 550993 |
| Alexa Fluor®647 rat anti-mouse CD206 | BD Pharmingen$^{TM}$ | 565250 |
| APC/Cyanine7 anti-mouse CD86 | Biolegend | 105030 |
| Anti-iNOS antibody | Abcam | ab15323 |
| Arginase-1 (D4E3M™) XP® rabbit monoclonal antibody | CST | 93668 |
| Phospho-mTOR (Ser2448) antibody | CST | 2971 |
| mTOR antibody | CST | 2972 |
| ULK1 (D8H5) rabbit monoclonal antibody | CST | 8054 |
| Phospho-ULK1 (Ser757) antibody | CST | 6888 |
| VPS33A polyclonal antibody | Proteintech | 16896-1-AP |
| Atg3 antibody | CST | 3415 |
| Atg7 (D12B11) rabbit monoclonal antibody | CST | 8558 |
| Atg16 antibody (E-10) | Santa Cruz | sc-393274 |
| Beclin-1 (D40C5) rabbit monoclonal antibody | CST | 3495 |
| SQSTM1/p62 antibody (BSA and azide-free) | Abcam | ab56416 |
| Atg12 (D88H11) rabbit monoclonal antibody | CST | 4180 |
| Atg5 (D5G3) rabbit monoclonal antibody | CST | 9980 |
| STX17 (E6R7P) rabbit monoclonal antibody | CST | 63022 |
| SNAP29 polyclonal antibody | Proteintech | 12704-1-AP |
| RAB7A (D95F2) XP® rabbit monoclonal antibody | CST | 9367 |
| Atg14 (D1A1N) rabbit monoclonal antibody | CST | 96752 |
| LC3A/B antibody | CST | 4108 |
| Transferrin receptor monoclonal antibody | Invitrogen | 13-6800 |
| Ferroportin polyclonal antibody | Invitrogen | PA5-22993 |
| Recombinant anti-ferritin antibody | Abcam | ab75973 |
| β-Actin (8H10D10) mouse monoclonal antibody | CST | 3700 |

**Sup. Fig. 1**

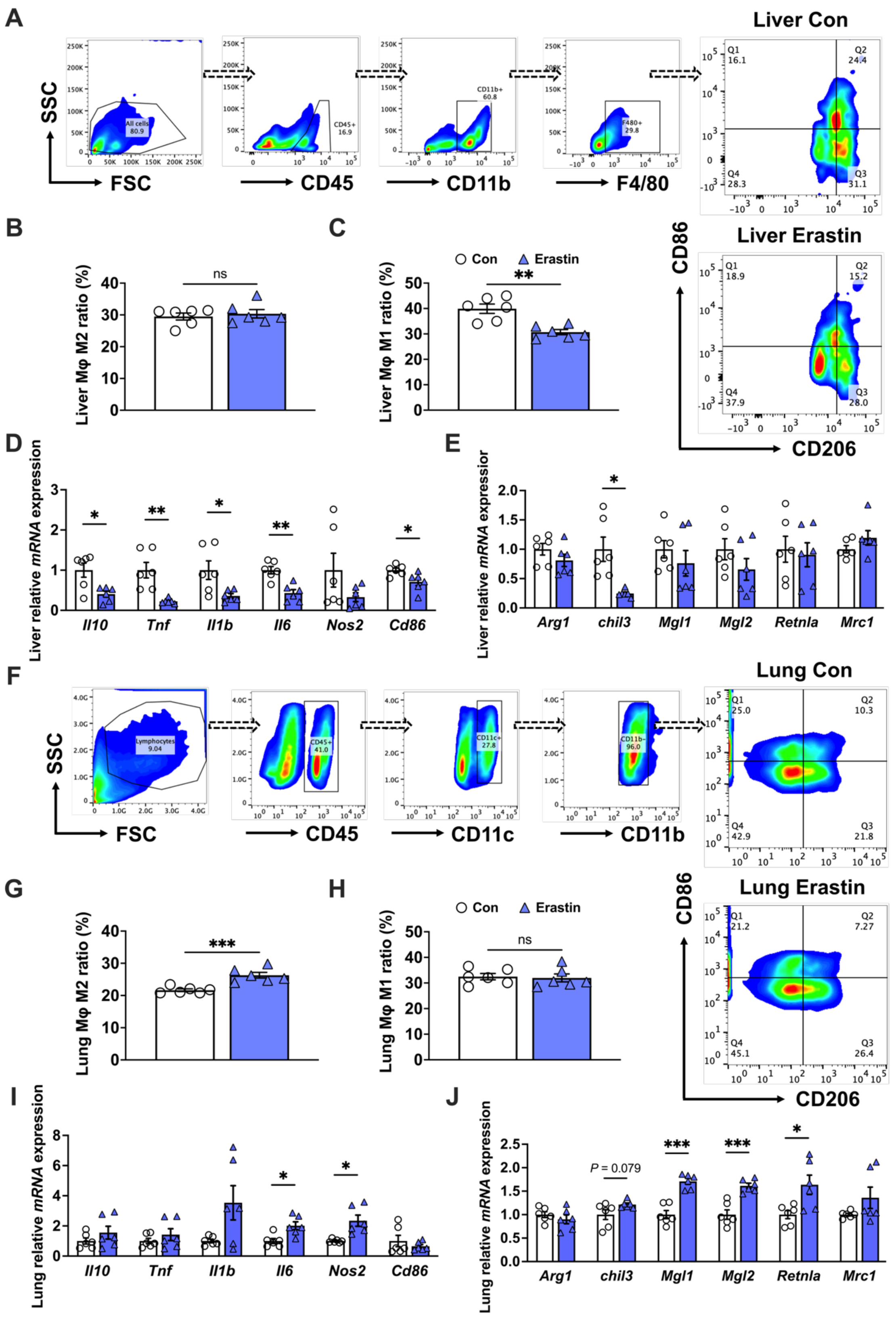


**Sup. Fig. 1.** Liver (kupffer cells) and lung (alveolar macrophages) M1 and M2 macrophages exhibit distinct responses to erastin-induced ferroptosis. mice were intraperitoneal injected with 25 mg/kg body weight of erastin or solvent (10% DMSO

+ 40% PEG300+ 5% Tween-80 + 45% physiological saline) for 2 days at 12 h intervals. Mice were anesthetized 6 h after the last injection. Flow cytometry was used to assess the proportions of M1 and M2 macrophage subtypes and the impact on M1 and M2 gene markers in macrophages derived from liver and lung tissues. (A) Flow cytometry gating strategy for liver macrophages in mice; (B) liver M1 macrophage ($CD45^+$, $CD11b^+$, $F4/80^+$, and $CD86^+$) ratio (n=6); (C) liver M2 ($CD45^+$, $CD11b^+$, $F4/80^+$, and $CD206^+$) macrophage ratio (n=6); (D) mRNA expression of M1-related markers (*Il10*, *Tnf*, *Il1b*, *Il6*, and *Nos2*) (n=6); (E) mRNA expression of M2-related markers (*Arg1*, *Chil3*, *Mgl1*, *Mgl2*, and *Retnla*) (n=6). (F) Flow cytometry gating strategy for lung macrophages in mice; (G) lung M1 macrophage ($CD45^+$, $CD11c^+$, $CD11b^-$, and $CD86^+$) ratio (n=6); (H) lung M2 ($CD45^+$, $CD11c^+$, $CD11b^-$, and $CD206^+$) macrophage ratio (n=6); (I) mRNA expression of M1-related markers (*Il10*, *Tnf*, *Il1b*, *Il6*, *Nos2* and *Cd86*) (n=6); (J) mRNA expression of M2-related markers (*Arg1*, *Chil3*, *Mgl1*, *Mgl2*, *Retnla* and *Mrc1*) (n=6). Liver Mφ = liver macrophages; Lung Mφ = lung macrophages. Student's t test was used to determine statistical significance, defined as * $P < 0.05$, ** $P < 0.01$, and *** $P < 0.001$.

**Sup. Fig. 2**

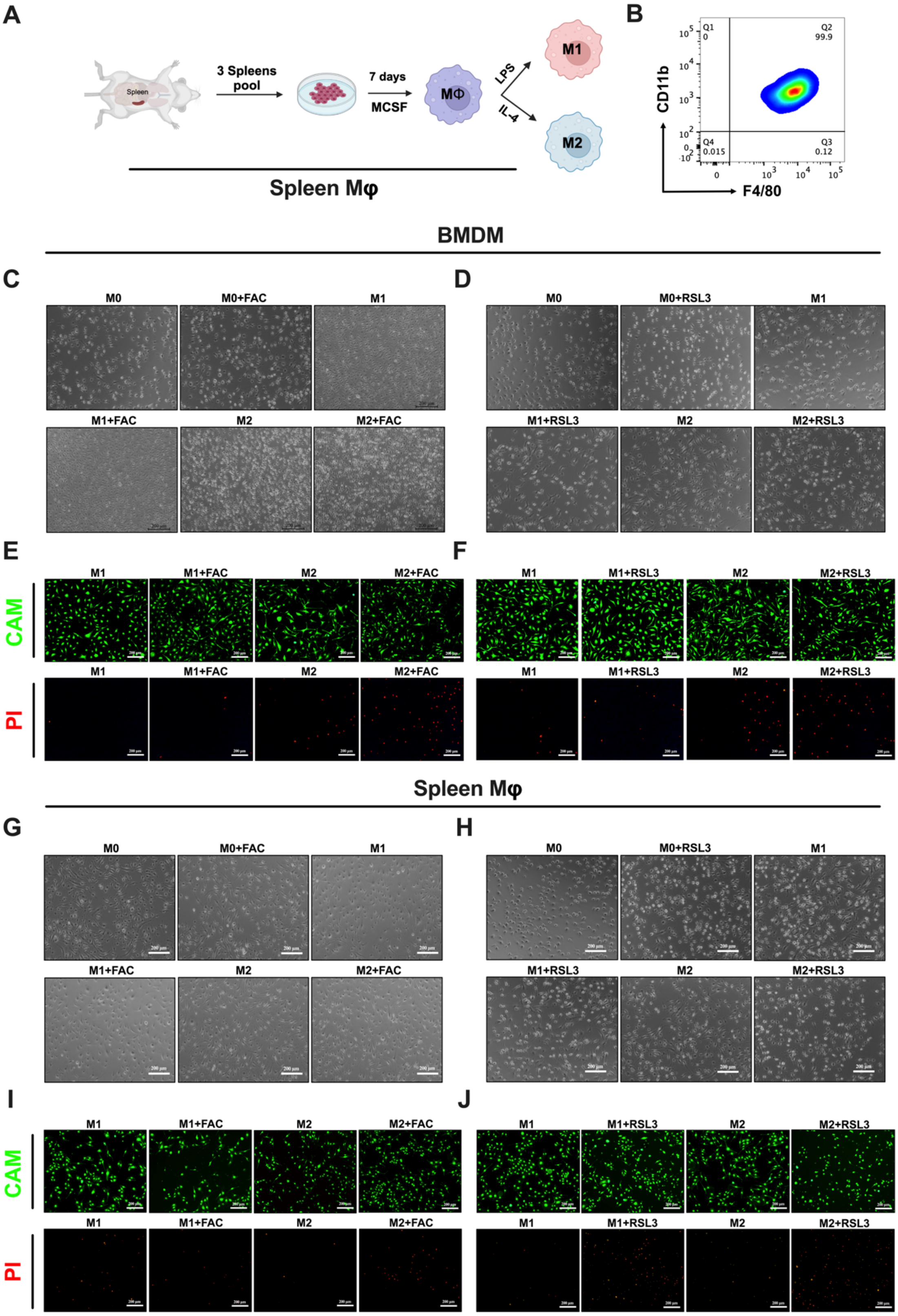


**Sup. Fig. 2.** Cell viability of polarized bone marrow-derived and splenic macrophages undergoing ferroptosis induced by ferric ammonium citrate (FAC) and RSL3. (A) Flowchart of the isolation, culturing, and polarization of splenic macrophages. (B) Identification of splenic macrophages as double positive for F4/80 and CD11b; (C and

D) Photomicrographs of BMDMs in different polarization states treated with FAC and RSL3. (E and F) Cell viability staining; CAM (green signal) indicates live cells, and PI (red signal) denotes dead cells. (G and H) Photomicrographs of splenic macrophages in different polarization states treated with FAC and RSL3. (I and J) Cell viability staining. CAM (green signal) indicates live cells, and PI (red signal) denotes dead cells. LPS = lipopolysaccharide; MCSF = macrophage colony stimulating factor; Spleen Mφ = splenic macrophages; CAM = cell adhesion molecule; PI = propidium iodide.

**Sup. Fig. 3**

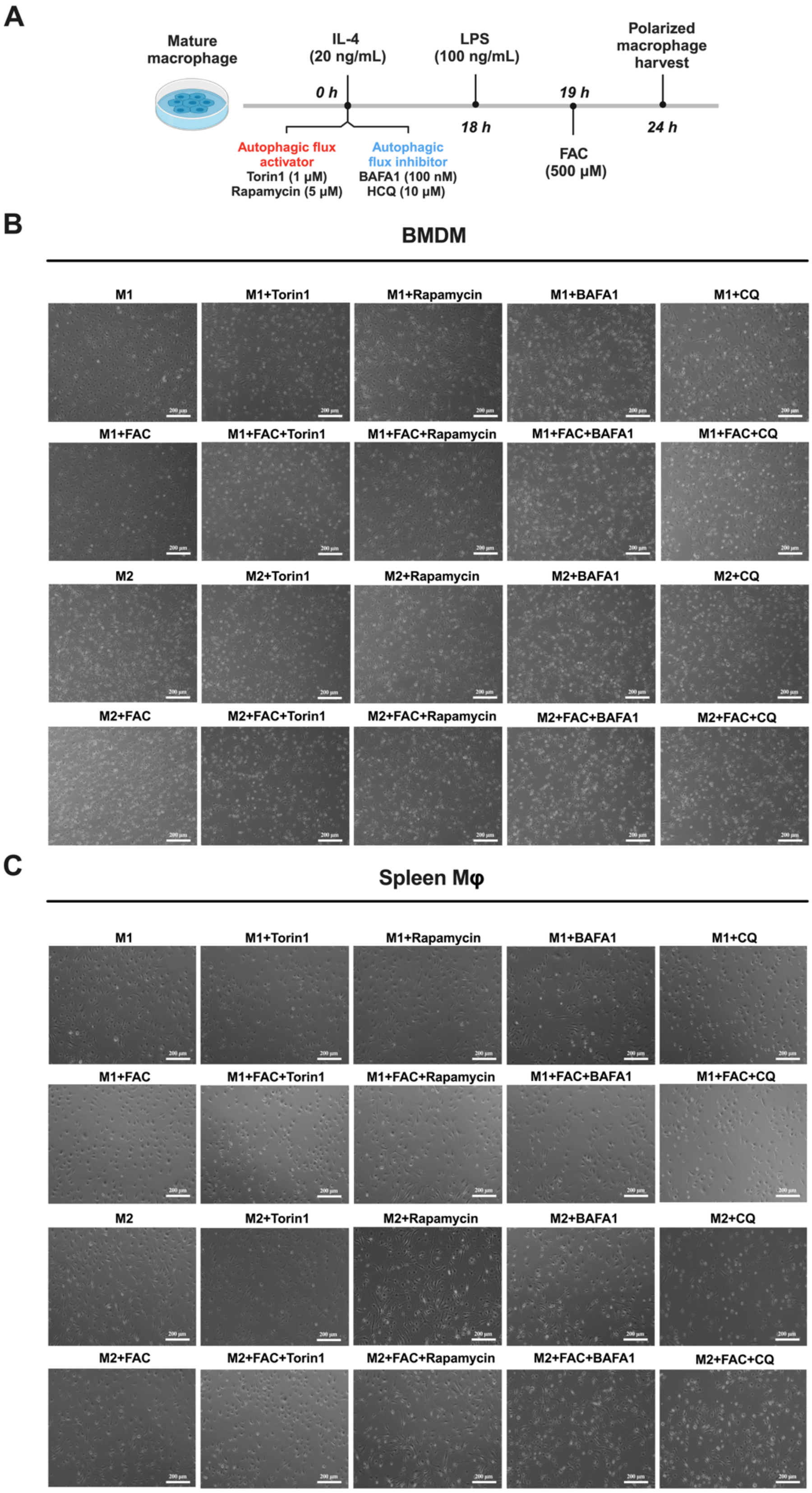


**Sup. Fig. 3.** BMDM and splenic macrophages responses to the autophagic flux activators and inhibitors. We treated BMDMs and splenic macrophages with autophagy activators (1 μM torin1 or 5 μM rapamycin) and autophagy inhibitors (100 nM BAFA1

or 10 μM HCQ). (A) Treatment dosage and procedure illustration; (B) Microscopic observation of BMDMs in different polarization states treated with autophagic flux activators and inhibitors; (C) Microscopic observation of splenic macrophages in different polarization states treated with autophagic flux activators and inhibitors. LPS = lipopolysaccharide; FAC = ferric ammonium citrate; Spleen Mφ = splenic macrophages.

**Sup. Fig. 4**

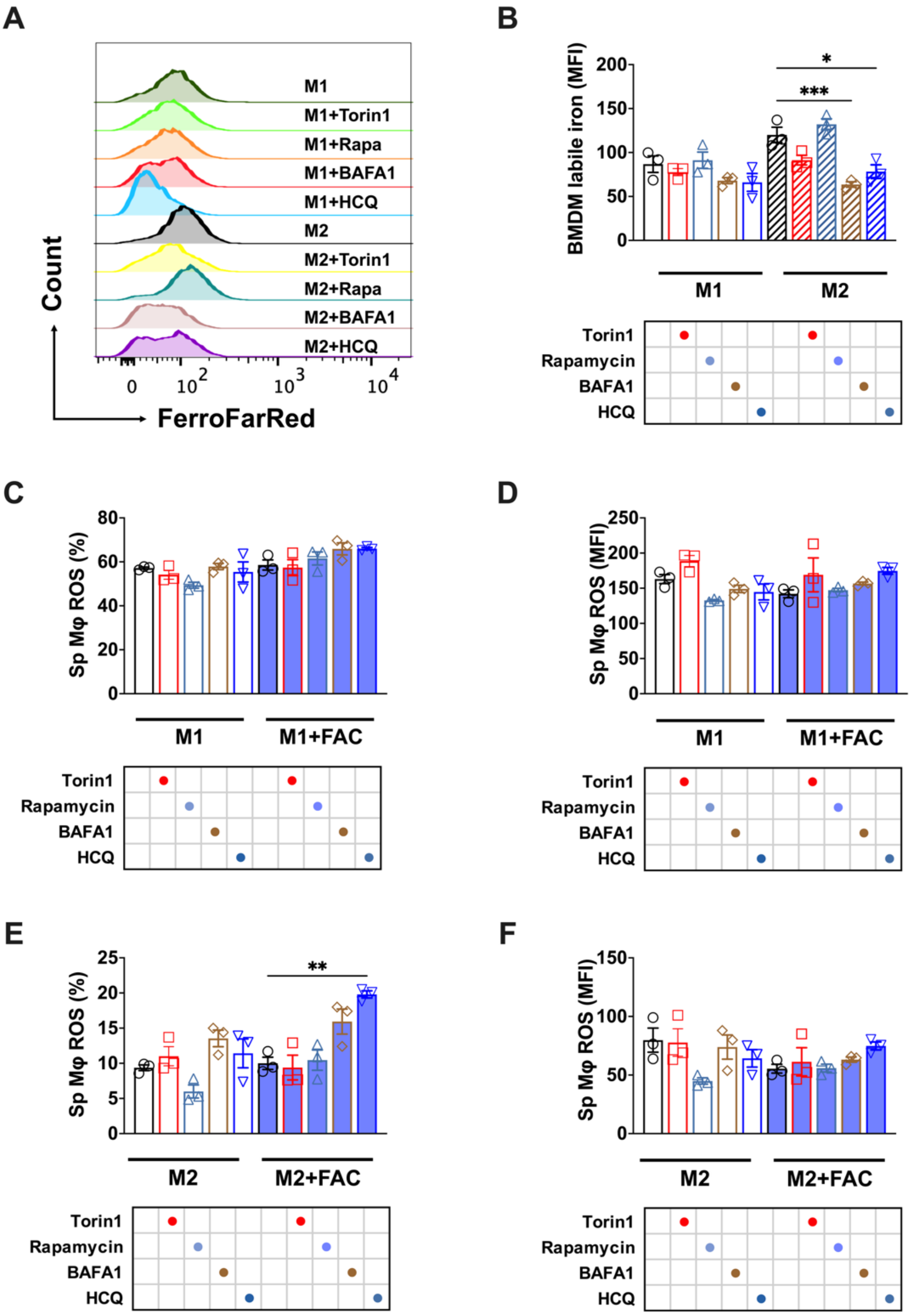


**Sup. Fig. 4.** The effects of autophagic flux regulation on labile iron and total ROS of BMDMs and splenic macrophages. (A) BMDM FerroFarRed labile iron pool flow cytometry histogram; (B) BMDM labile iron MFI (n=3); (C to F) ROS-positive rate and MFI of splenic macrophages (n=3). ROS = reactive oxygen species; Sp Mφ = splenic macrophages. One-way ANOVA (Tukey) was used to determine statistical significance, defined as * $P < 0.05$, ** $P < 0.01$, and *** $P < 0.001$.

**Sup. Fig. 5**

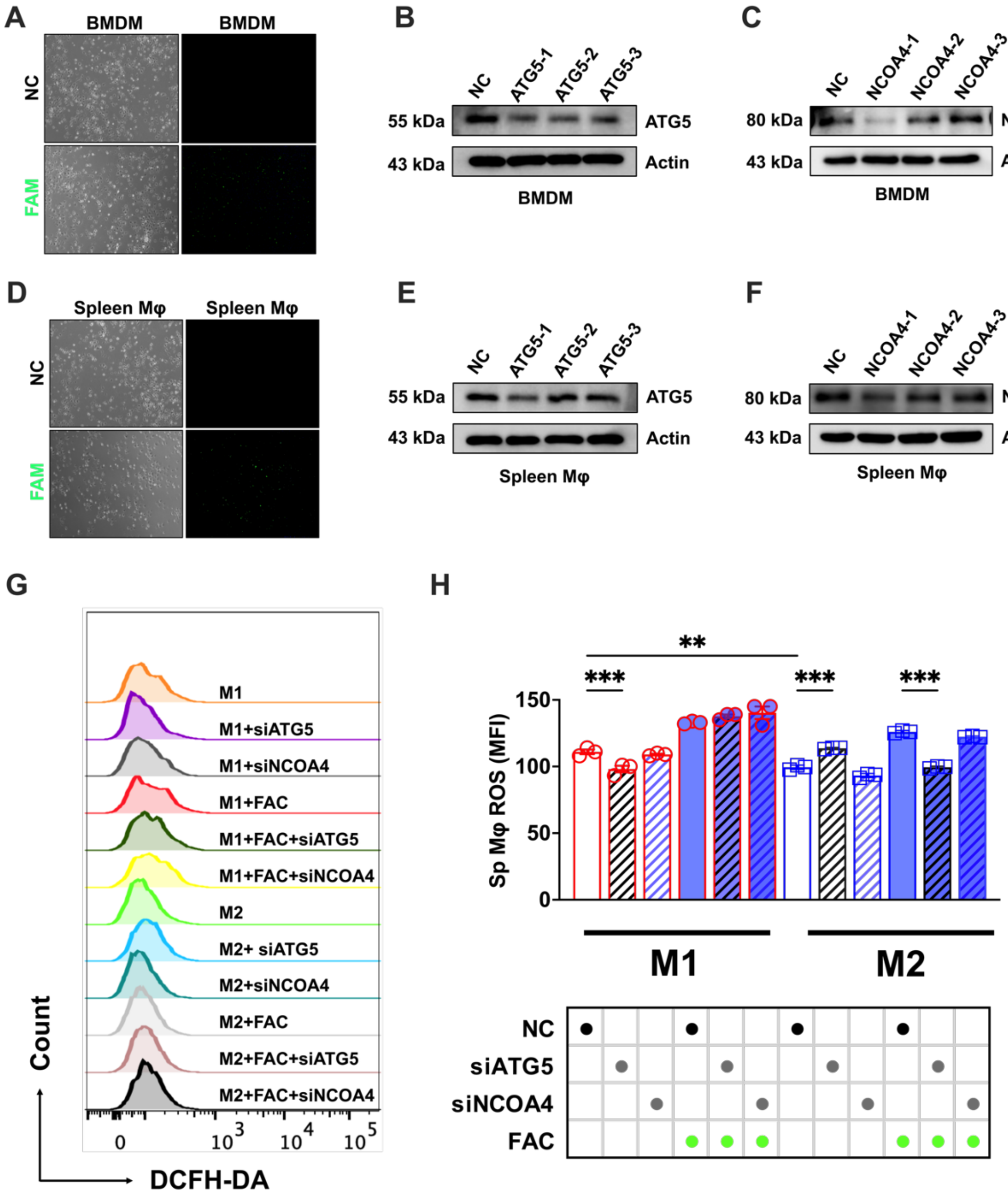


**Sup. Fig. 5.** Identification of the transfection efficiency and knockdown efficacy of ATG5 and NCOA4 siRNA. (A) BMDM siRNA transfection efficiency, with FAM fluorescently labeled siRNA; (B to C) Western Blot identification of ATG5 and NCOA4 knockdown in BMDMs; (D) identification of siRNA transfection efficiency in splenic macrophages, with FAM fluorescently labeled siRNA; (E to F) Western Blot identification of ATG5 and NCOA4 knockdown in splenic macrophages. Splenic macrophages were subjected to knockdown of key ferritinophagy proteins using siATG5 and siNCOA4, and the levels of ROS were measured under a gating strategy using CD206 (M1) and CD86 (M2). (G and H) RAW264.7 cell DCFH-DA total ROS flow cytometry histogram and ROS MFI. ATG5 = autophagy-related 5; NCOA4 = nuclear receptor coactivator 4; ROS = reactive oxygen species; Sp Mφ = splenic macrophages. One-way ANOVA (Tukey) was used to determine statistical significance, defined as * P < 0.05, ** P < 0.01, and *** P < 0.001.

**Sup. Fig. 6**

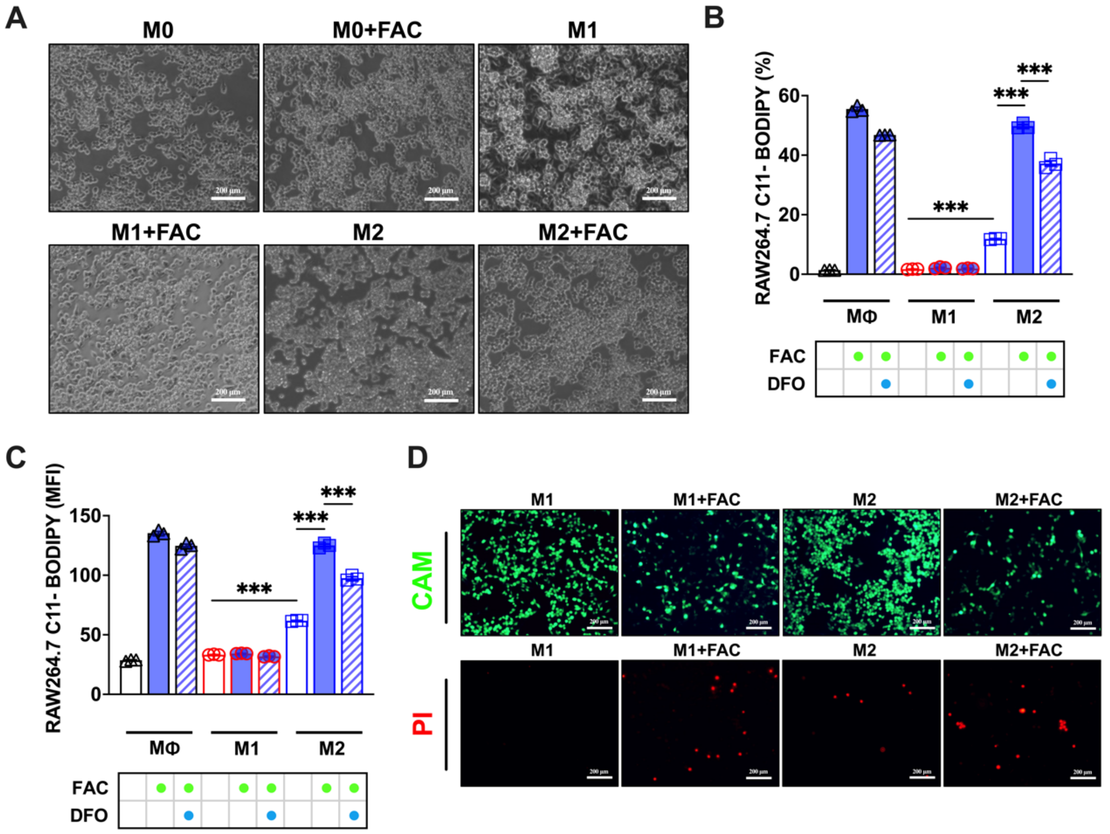


**Sup. Fig. 6.** RAW264.7 M1 and M2 macrophages shared a similar ferroptosis sensitivity pattern as the bone marrow macrophages. (A) Microscopic observation of RAW264.7 cells in different polarization states treated with FAC and RSL3; and (B and C) percentage of polarized RAW264.7 cells treated with FAC positive for lipid peroxidation and the MFI (n=3). (D) Cell viability staining. CAM (green signal) indicates live cells, and PI (red signal) denotes dead cells. FAC = ferric ammonium citrate; RSL3 = RAS-selective lethal 3. One-way ANOVA (Tukey) was used to determine statistical significance, defined as * $P < 0.05$, ** $P < 0.01$, and *** $P < 0.001$.

**Sup. Fig. 7**

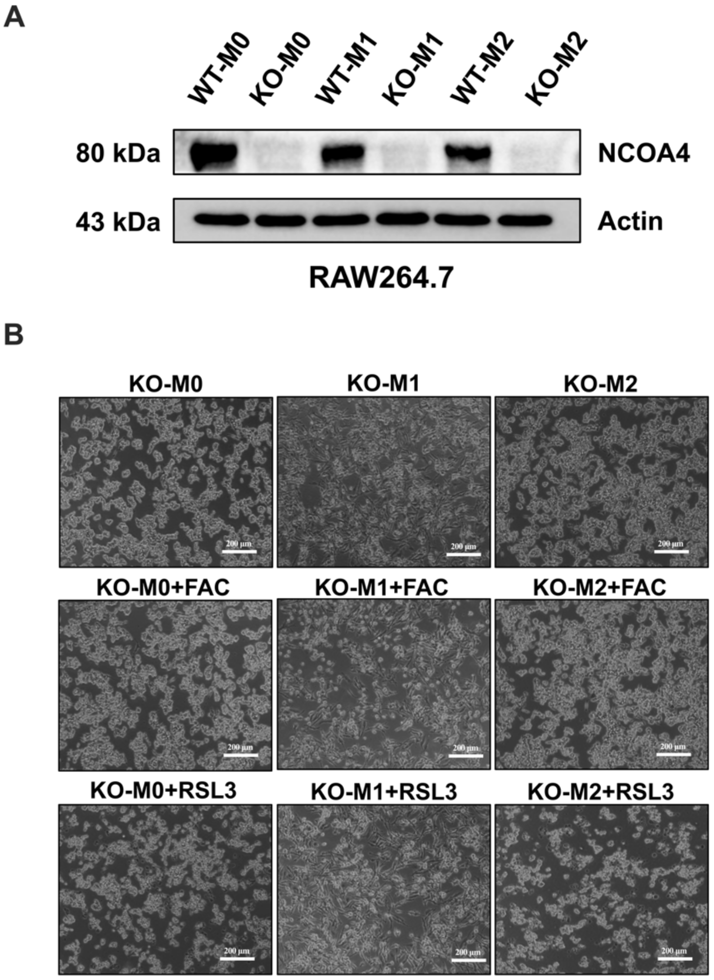


**Sup. Fig. 7.** NCOA4 knockout out confirmation. (A) Western blot bands for NCOA4 in polarized RAW264.7 wild-type and knockout cell lines; (B) Photomicrographs of NCOA4-KO RAW264.7 cells in different polarization states treated with FAC and RSL3. FAC = ferric ammonium citrate; RSL3 = RAS-selective lethal 3; NCOA4 = nuclear receptor coactivator 4.

**Sup. Fig. 8**

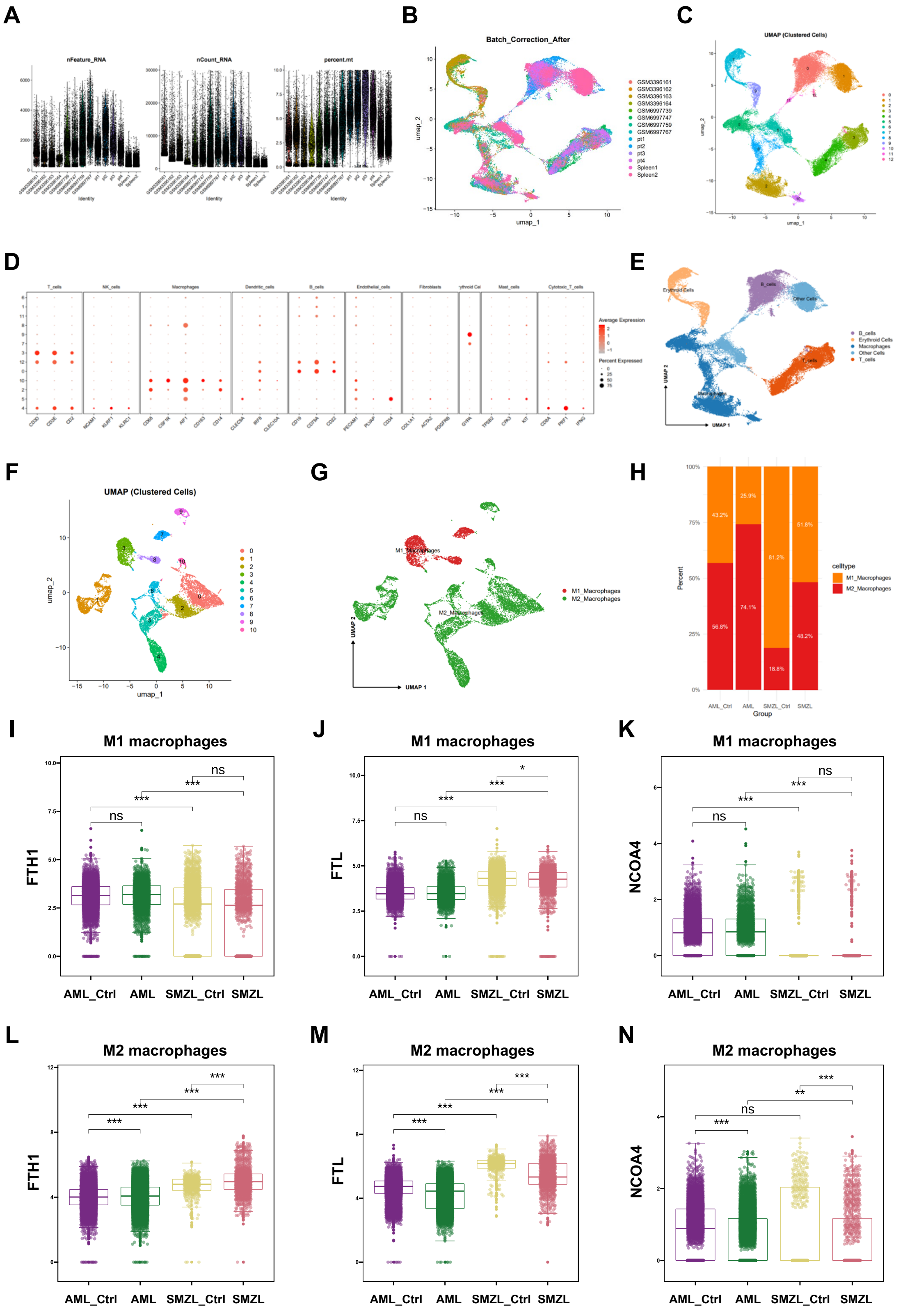


**Sup. Fig. 8.** Single cell transcriptomic profiling of macrophages from human spleen and bone marrow across healthy and diseased states. (A) Quality control metrics of single-cell RNA-seq datasets, showing nFeature_RNA, nCount_RNA, and percent mitochondrial gene content (percent.mt) for each cell across all samples. Cells with

nCount_RNA > 500, nCount_RNA < 30,000, nFeature_RNA > 200, nFeature_RNA < 8,000, percent.mt < 10 were retained; (B) Batch effect correction across different samples using Harmony integration method; (C) UMAP visualization showing unsupervised clustering of all cells; (D) Dot plot showing the expression of canonical marker genes used for cluster annotation; (E) Annotated cell types based on marker gene expression. Macrophages were specifically identified for further analysis; (F) Re-clustering of macrophage populations reveals subclusters; (G) Re-annotation of macrophage subpopulations, identifying M1 and M2 macrophages based on marker gene expression; (H) Proportional distribution of M1 and M2 macrophages across different experimental groups (AML_Ctrl, AML, SMZL_Ctrl, and SMZL); (I to N) Box plots showing expression levels of *Fth1*, *Ftl1*, and *Ncoa4* in M1 and M2 macrophages across bone marrow and spleen under normal and pathological conditions. Ncoa4 = nuclear receptor coactivator 4; Fth1 = ferritin heavy chain 1; Ftl1 = ferritin light chain; AML_Ctrl = healthy human bone marrow sample; AML = acute myeloid leukemia bone marrow sample; SMZL_Ctrl = healthy human spleen sample; SMZL = splenic marginal zone lymphoma sample. One-way ANOVA (Tukey) was used to determine statistical significance, defined as * $P < 0.05$, ** $P < 0.01$, and *** $P < 0.001$.

**Sup. Fig. 9**

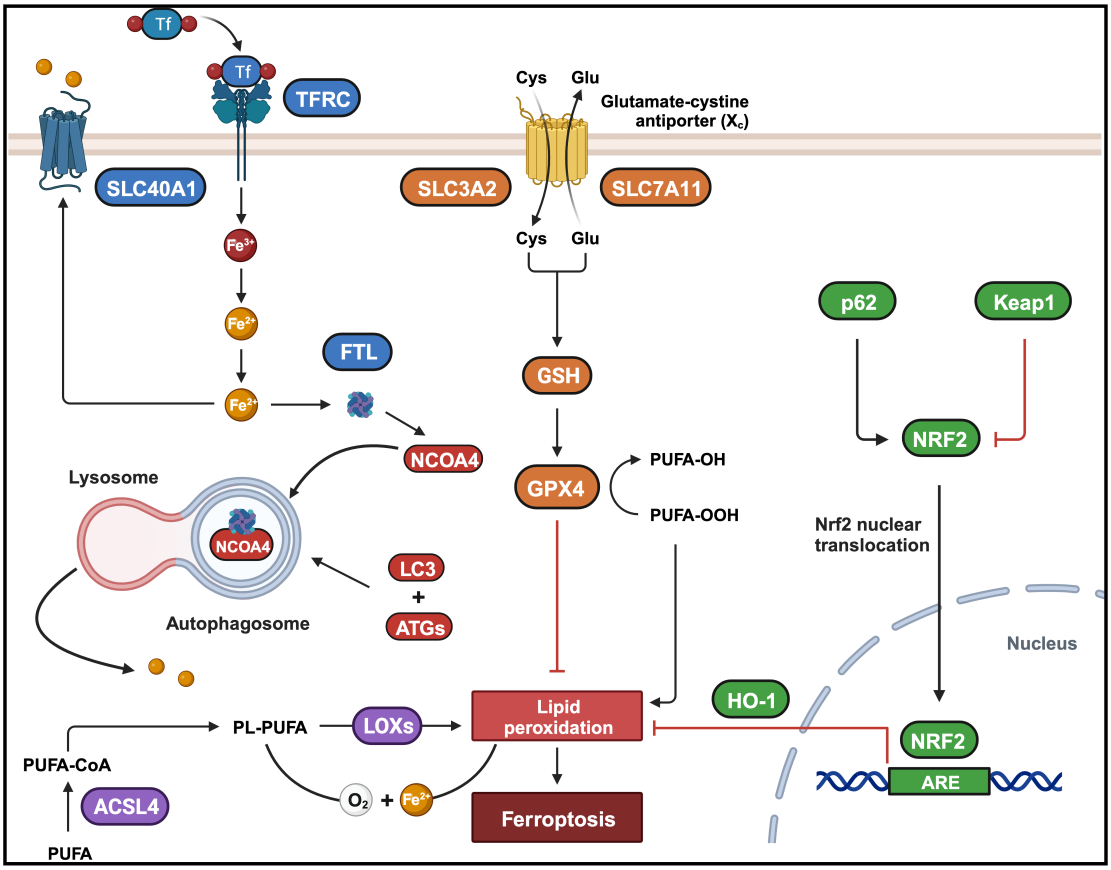


**Sup. Fig. 9.** The primary signaling pathways influencing the ferroptosis process. Five key pathways influence ferroptosis: NRF2-centered antioxidative signaling (green); cysteine transport and the GSH/GPX4 system (orange), which inhibits lipid peroxidation; iron metabolism (blue) and ferritinophagy pathways (red), which regulate ferroptosis through *Fenton reaction* substrates and labile iron levels; and lipid metabolism (purple), which is critical because of the reliance of ferroptosis on unsaturated fatty acid oxidation.

## Supplementary Methods

### RAW264.7 NCOA4 knockout cell line

A RAW264.7 NCOA4 knockout cell line was obtained from Haixing Bioscience. To construct the knockout cell line, the cells were first verified for contamination and proliferation. Next, guide RNA (gRNA) and Cas9 protein were synthesized according to the design scheme. Cells are then transfected using the Neon™ transfection system with the gRNA and Cas9 RNP complex. After electroporation, cells were digested and counted to establish monoclonal cultures. PCR was performed to identify gRNA target sites and knockout bands in clones, followed by sequencing to verify successful clones. Finally, positive clones were expanded for further applications.

### Fluorescence staining with calcein-AM and propidium iodide

To assess cell viability and cell death, calcein-AM (CAM) (206700, Sigma-Aldrich, St. Louis, MO, United States) and propidium iodide (PI) (P4170, Sigma-Aldrich, St. Louis, MO, United States) staining was performed. Cells were seeded in 24-well plates and allowed to adhere overnight. Post-treatment, cells were washed with PBS and stained with CAM and PI. Specifically, CAM (2 μM) and PI (4 μM) were diluted in PBS and added to the cells. The cells were incubated at 37°C for 15 min in a $CO_2$ incubator to allow for sufficient staining. CAM stains viable cells by emitting green fluorescence upon cleavage by intracellular esterases, while PI penetrates and stains the nuclei of dead cells with red fluorescence.

### Transcriptomic data acquisition and analysis

Total RNA was extracted using TRIzol reagent (15596026, Invitrogen, Thermo Fisher Scientific, Waltham, MA, USA). RNA quantity, purity, and integrity were evaluated with a NanoDrop spectrophotometer (Thermo Scientific). Three micrograms of RNA were used for library construction. To begin, mRNA was isolated using magnetic beads conjugated with poly-T oligonucleotides. Fragmentation of mRNA was carried out in a proprietary Illumina buffer containing divalent cations at elevated temperatures. First-strand cDNA synthesis was performed with random primers and SuperScript II, followed by second-strand synthesis using DNA polymerase I and RNase H. The resulting cDNA fragments were end-repaired to produce blunt ends, and enzymes were removed. A-tailing was then conducted at the 3′ ends, followed by ligation of Illumina paired-end adapters. Library fragments were size-selected (400–500 bp) and purified using the AMPure XP system (Beckman Coulter, Beverly, CA, USA). Adapter-ligated fragments were amplified via 15 cycles of PCR using Illumina's PCR primer cocktail. The final libraries were purified again with AMPure XP beads and quantified using a High Sensitivity DNA assay on the Agilent Bioanalyzer 2100 system. Sequencing was carried out on the Illumina NovaSeq 6000 platform.

### Autophagic flux monitoring using a dual-labeled adenovirus (mRFP-GFP-LC3)

BMDMs were cultured in 12-well plates after passage until they reached a confluence of 40%–50%. The cells were transfected with 2 μL of mRFP-GFP-LC3 adenovirus and

incubated for 24 h. Following three washes with PBS, the cells were subjected to M1 or M2 macrophage polarization media and cultured for an additional 24 h. Fluorescent imaging was conducted using an inverted fluorescence microscope (Leica DMI 4000B, USA).

**FTH1 knockdown by shRNA adenovirus transduction**

Short hairpin RNA (shRNA) targeting FTH1 and negative control (NC) shRNA were designed and constructed by Hanbio Biotechnology with the following sequences: negative control shRNA (top strand), GATCCGTTCTCCGAACGTGTCACGTAATTCAAGAGATTACGTGACACGTTCGGAGAATTTTTTC; negative control shRNA (bottom strand), AATTGAAAAAATTCTCCGAACGTGTCACGTAATCTCTTGAATTACGTGACACGTTCGGAGAACG; FTH1 shRNA (top strand), TCGAGGACTTCATTGAGACGTATTATTTCAAGAGAATAATACGTCTCAATGAAGTCTTTTTTA; and FTH1 shRNA (bottom strand), AGCTTAAAAAAGACTTCATTGAGACGTATTATTCTCTTGAAATAATACGTCTCAATGAAGTCC.

Cells were seeded in 6-well plates at 60%–70% confluency and incubated overnight. The next day, the medium was removed, and cells were washed with PBS. The transduction mix, containing the shRNA adenovirus (NC or FTH1) and serum-free medium, was prepared with an MOI of 100. Cells were incubated with the virus for 6 h at 37°C in 5% $CO_2$, after which the medium was replaced with fresh complete growth medium. Following 48 h of incubation, cells were collected for analysis.

**ATG5 and NCOA4 knockdown**

Mouse ATG5- and NCOA4-targeting siRNAs (sense and antisense) and a non-targeting control siRNA were sourced from Sangon Biotech. BMDMs were transfected with the siRNAs using Lipofectamine 3000 reagent (L3000001, Thermo Fisher Scientific, Waltham, MA, United States). On day 0, BMDMs were cultured in 10-cm dishes. On day 4, cells were washed with PBS and refreshed with DMEM supplemented with 10% FBS (without antibiotics or M-CSF). A two-tube setup was used, in which tube 1 contained 1 mL of opti-MEM (31985062, Gibco, Thermo Fisher Scientific, Waltham, MA, United States) and 74 μL of Lipofectamine 3000, while tube 2 contained 1 mL of opti-MEM and 74 μL of 10 μM siRNA (separate tubes for each siRNA). After briefly mixing each tube, the contents of tube 2 were added to tube 1 and incubated for 5 min at RT. The formed transfection complexes were then applied dropwise (2 mL per 10-cm dish) onto the cells. On day 5, cells were washed with PBS, and BMDM medium with 10% FBS, 1% PS, and M-CSF was added to the cells. On day 6, cells were collected, counted, and replanted into smaller dishes according to the experimental plan. Finally, on day 7, the cells were used in experiments.

The siRNA sequences were as follows (all 5’-3’): NCOA4 sense, CGAUCUCAUCUAUCAGCUUAATT; *NCOA4* antisense, UUAAGCUGAUAGAUGAGAUCGTT; ATG5 sense, CCUUGGAACAUCACAGUACAUTT; *ATG5* antisense,

AUGUACUGUGAUGUUCCAAGGTT; NC sense, UUCUCCGAACGUGUCACGUTT; and *NC* antisense, ACGUGACACGUUCGGAGAATT.

**Single cell RNA sequencing data processing and macrophage subtype analysis**

Single cell RNA sequencing datasets were obtained from the Gene Expression Omnibus (GEO) database, including normal spleen (GSE134355), splenic marginal zone lymphoma (SMZL) (GSE286927), acute myeloid leukemia (AML) (GSE120221), and its corresponding healthy control (GSE223844). Raw gene expression matrices were processed using the Seurat package in R. Quality control was performed by retaining cells with 500–30,000 total RNA counts, 200–8,000 detected genes, and <10% mitochondrial gene content. To eliminate batch effects across datasets, Harmony integration was applied after normalization. Principal component analysis (PCA) was conducted on the top 2,000 highly variable genes prior to integration. Dimensionality reduction was carried out using Uniform Manifold Approximation and Projection (UMAP), and clustering was performed using the FindNeighbors and FindClusters functions in Seurat. Cell types were annotated based on canonical marker genes with reference to PanglaoDB and CellMarker databases. Macrophage populations were identified and subjected to secondary clustering to analyze subpopulation characteristics. M1 and M2 polarization states were evaluated using gene set scoring based on established marker gene sets.
